# A mixed methods systematic review of the impact of paediatric mental health liaison services on children and young people’s mental and physical health, stakeholder experience, and service-level outcomes

**DOI:** 10.1007/s00787-025-02815-5

**Published:** 2025-07-15

**Authors:** Miriam Avery, Sue Kirk, Steven Pryjmachuk

**Affiliations:** 1 University of Manchester and Greater Manchester Mental Health NHS Foundation Trust, Greater Manchester, England; 2 University of Manchester, Greater Manchester, England

**Keywords:** Liaison, Psychiatry, Psychology, Paediatric, Integrated care, Systematic review

## Abstract

Children and young people (CYP) with physical and mental health needs commonly present to acute hospitals. Although evidence shows liaison services providing mental health assessment and intervention to adult acute hospitals users improve outcomes, there is no evidence review for Paediatric Mental Health Liaison (PMHL). A mixed-methods, integrative, systematic review, using Joanna Briggs Institute (JBI) guidance, was conducted to identify evidence for PMHL service impacts on stakeholder experience and health and service level outcomes in the acute hospital setting.

Keyword searches of relevant databases including Medline and PsycINFO plus citation searching identified 10,111 records; 415 papers were eligible for full-text screening. 53 papers describing mental health services/interventions provided to CYP aged 0–25 in acute hospital settings and reporting data on health and service-level outcomes, or stakeholder experiences were included. Service description, outcomes, and stakeholder experience data were extracted from included papers. Quantitative findings were ‘qualitized’ and analysed thematically. Quality appraisal used JBI tools.

Studies were mostly cross-sectional (*n* = 27) or quasi-experimental (*n* = 13), from the US (*n* = 28) or England (*n* = 11). Service descriptions ranged from multidisciplinary teams covering entire hospitals to single mental health professionals integrated into one physical health department. Presence of PMHL services improve length of stay and healthcare costs/utilization, but evidence has methodological limitations. Evidence for PMHL services’ impact on health outcomes is also positive but weak. PMHL services are valued by physical healthcare staff and parents/carers, but CYP views were underrepresented. Further work needs to establish outcomes important to CYP and families.

Prospero Registration Number: CRD42022383611

**Supplementary Information:**

The online version contains supplementary material available at 10.1007/s00787-025-02815-5.

## Introduction

Children and Young People’s (CYP) mental health is a global public health priority. The World Health Organisation estimates that 10% of CYP worldwide experience a mental disorder, although most will not seek help or receive appropriate care [1]. Recent data suggests increasing use of generalist settings, likely due to the inability of community child and adolescent mental health services (CAMHS) to cope with the uptick in referrals over the past few years and an upwards trend in the numbers of CYP presenting to Emergency Departments (ED) and being admitted to paediatric wards for a primary mental health reason [2–5].

Primary mental health reasons include CYP who attempt suicide, or experience conditions with both a physical and psychological component, e.g. self-harm, eating disorders, functional somatic symptoms (FSS) [6–8]. CYP with long term physical conditions (LTCs) also fall into this category, being up to four times more likely to experience psychiatric symptoms than their peers [9]. Care for CYP in these groups requires an approach which addresses physical and psychological issues simultaneously [10–13]. However, acute hospital professionals may not have the training, skills, or knowledge to identify or appropriately manage psychological needs [14]. Consequently, this group of CYP are at risk of inappropriate, inadequate, or unnecessary investigations and treatments, with delayed referral to specialist CAMHS able to provide treatment for more complex or persistent mental health difficulties.

One potential solution to this problem is the development of service models which integrate mental and physical health services, such as mental health liaison. In England and the United States (US), mental health liaison for CYP is a less well-developed sub-specialty than for adults [15–17] and is more likely to be provided on an ad-hoc basis or by external providers, rather than by a dedicated team located in the acute hospital. The term ‘paediatric mental health liaison’ (PMHL) is therefore broadly applied to include any service or intervention provided to CYP aged 0–25 by a mental health professional or team of primarily mental health professionals of any discipline working in an acute hospital setting.

Compared to adult mental health liaison, where there is a wealth of evidence looking at the impact of services, (e.g., the systematic review of consultation liaison psychiatry by Wood and Wand [18]), the evidence for the impact of PMHL services has not been systematically reviewed. Given the increasing numbers of CYP presenting to the acute hospital requiring both physical and mental health care [8, 19], a robust review of evidence is needed to establish what is currently known about these services and the factors supporting the development of effective and accessible approaches to healthcare for CYP with co-occurring physical and psychological difficulties.

A mixed methods review approach has been chosen to bring together the findings of effectiveness (quantitative evidence) and patient, family, staff experience (qualitative evidence) to enhance their usefulness to decision-makers [20]. Both elements are crucial in developing clinically effective and efficient PMHL services which are acceptable to CYP, families, and staff.

### Review question

What is known about the impact of PMHL services in EDs, paediatric wards, and acute paediatric outpatient settings on stakeholder experience and health and service level outcomes? Specifically,

- What are the different models of PMHL described in the international literature?
- How do CYP, families/carers, and staff experience using or providing PMHL services?
- What impacts do different PMHL service models have on health and service-level outcomes?

The review aims, therefore, are:

1. To summarise the international literature describing the nature and function of PMHL services.
2. To evaluate the international literature evidencing experiences of service users and service providers of PMHL services.
3. To evaluate the international literature evidencing the impact of PMHL service delivery on CYP health and health service outcomes.

## Methods

### Search strategy

Search terms were developed using the ‘PICOS’ framework, in line with the Joanna Briggs Institute (JBI) mixed-methods review process [21]. As the review question was comprised of three concepts– ‘paediatric’, AND ‘mental health’ AND ‘liaison’– a variety of search terms were used to identify papers representing all three concepts (See Appendix 1). Databases searched were Medline, PsycINFO, PsycBooks, AMED, Embase, HMIC, EBM Reviews, Social Policy and Practice, CINAHL, PubMed, Prospero and ClinicalTrials.Gov. Reference lists of papers that met the inclusion criteria were also searched.

Inclusion and exclusion criteria were mapped to PICOS (Table 1). No study design or outcome limitations were imposed due to the lack of previous systematic reviews on the topic, however papers that simply described PMHL services were excluded to retain the focus on rigorously determined outcome or experience data. Date was limited to 1991 in reference to a commonly cited paper which appears to be the first published description of a paediatric liaison psychiatry service in England [22]. Primary care or community health settings were excluded to maintain focus on interventions and services for CYP in the acute general hospital setting. Studies concerned with changes to service provision or care-experience due to the Covid-19 pandemic were excluded to retain focus on the impact of usual PMHL working practices.

**Table 1:** Inclusion and exclusion criteria

	Inclusion	Exclusion
Population	Paper refers to intervention or service provided to CYP aged 0–25 years.	Papers referring to service users age 25 + or data regarding 0–24 not separable from other age ranges, or mean age greater than 25.
Intervention	Multidisciplinary mental health-focused service or single mental health professional providing any mental health intervention.	Where research focus is: evaluating the method of delivery alone, e.g. telehealth, without any reference to outcomes (as defined below) changes made or experiences of care during/as a result of Covid-19 pandemic interventions not provided by a mental health professional or the person providing intervention does not usually work in that setting (e.g. research assistants)
Context	Acute general hospital settings– EDs, paediatric wards, paediatric outpatient clinics.	Inpatient psychiatric settings primary care or community mental health settings. Intervention or service provided has no relation to a service user presentation or admission to the acute hospital, e.g., provision of tele-psychiatry to a primary care physician.
Outcome	Included papers must have extractable data related to at least one of the following: stakeholder experience of providing or using the service or intervention any measurable health-related outcome (e.g., anxiety score, quality of life measure) any measurable service level outcome (e.g., length of stay, cost of treatment/care)	Not possible to (a) identify or (b) extract data regarding stakeholder experience or measurable outcomes
Study Type	Primary research including: qualitative, mixed-methods, quantitative studies Formal service evaluations with clearly defined methods and measurable outcomes.	Purely descriptive studies with no measurable outcomes; protocols, posters and/or conference proceedings; secondary research, e.g. systematic reviews; discursive/opinion articles; dissertations and theses; audits; prevalence, incidence, epidemiological studies; descriptive case studies/series. This ensured papers included had sufficient data to allow links to be made between described service models and outcomes.

**Fig. 1:**
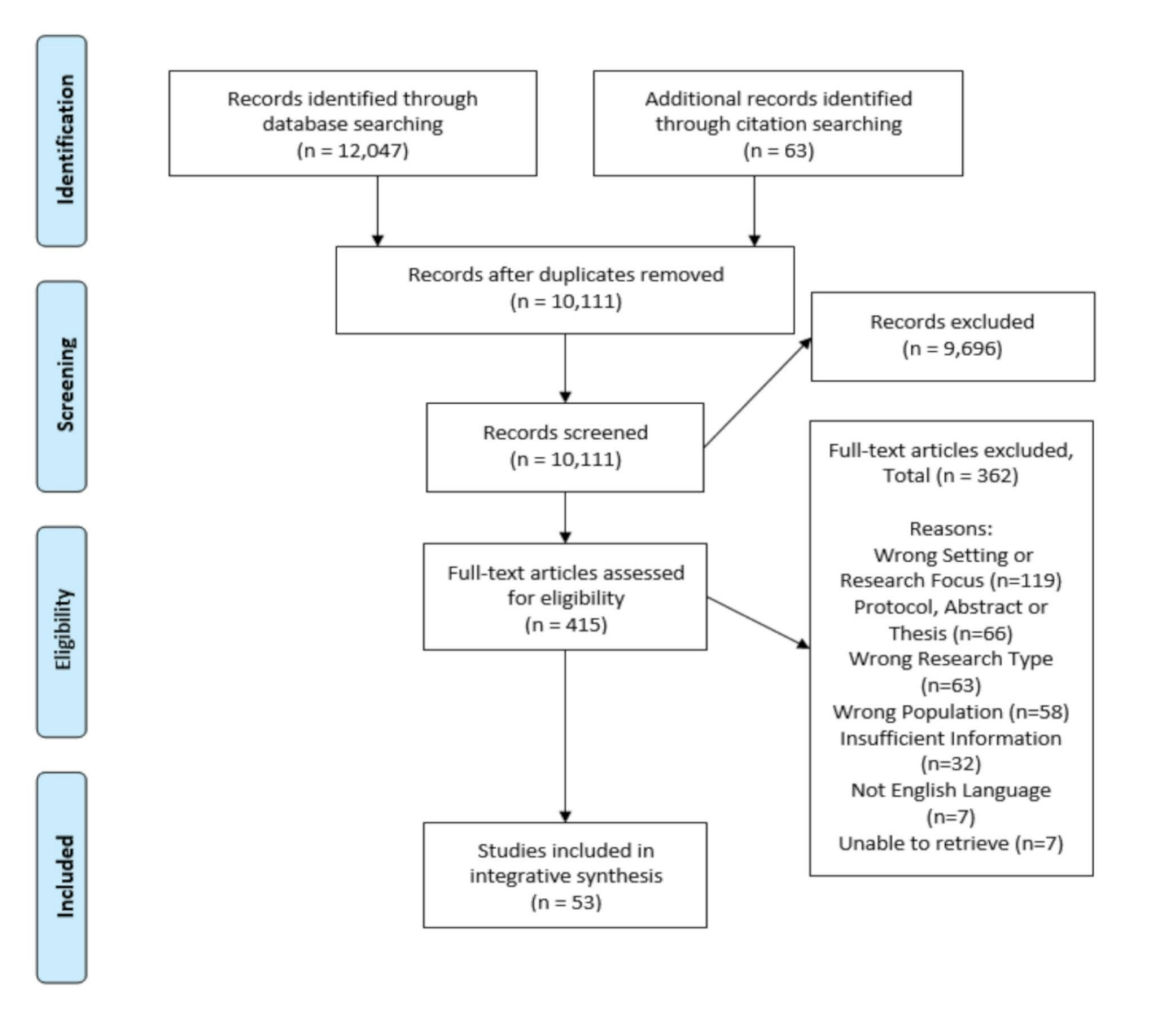
PRISMA diagram [26]

12,047 citation records were retrieved from the database searches, all conducted in February 2024. These were downloaded into Endnote 20 [23] for deduplication. Deduplicated records were then uploaded into the review management program, Covidence [24]. Following deduplication, and an additional 63 records identified from citation searching, a total of 10,111 records remained for title and abstract screening by the review team. 415 records were identified for full text screening which were independently screened by two reviewers. Disagreements were resolved by discussion with a third reviewer. 53 papers reporting 53 studies ultimately met the inclusion criteria. Five records were sought but were not located, despite efforts by the lead author; these were all due to no response from named authors or inter-library loan requests (See Fig. 1).

### Data extraction 

For the 53 included studies, all data from key categories (author; date; year; sample details; research design; intervention/research question details; key findings) were extracted by the lead author into an Excel [25] spreadsheet (see Appendix 2 for an extraction overview). A random selection of ten papers (18%) was blindly extracted by another member of the review team and then compared to the original. Extractions of a further ten papers were checked by two members of the review’s Young Person’s Advisory Group (YPAG), who met with the lead author to discuss the information extracted for each category in comparison to that contained in the original paper, and to ascertain whether there were any differences in understanding of what should be extracted. There was a high level of agreement between all extractors and during extraction checking discussions, with very few papers identified where authors did not agree with content of data extraction. Any discrepancies were minor and easily resolved. No data were found to be missing during the extraction process.

### Quality appraisal methods

In line with the JBI mixed method review approach, each paper was appraised using the relevant JBI quality appraisal tool aligned to its research design [27]. The lead author read and re-read each paper alongside the relevant JBI tool, identifying information which was then utilised to answer each question contained within the tool (or to confirm that information was not present or not relevant in the paper). For example, the qualitative tool asks; ‘Is there a statement locating the researcher culturally or theoretically?’ If an explicit description of the researcher’s approach, or professional background was present, this would elicit a ‘yes’. If this information could be implied from context or other information but was not stated, this would elicit an ‘unclear’. If this information was neither implied or explicit, this would be a ‘no.’ Similarly, the cross-sectional tool asks ‘were the study subjects and setting described in detail?’ In this case, if there was both a statement locating the study (e.g. in paediatric ward, across all areas of a general hospital, ED only), and any relevant team/intervention studied in detail, plus a table clearly setting out key participant characteristics including demographics, diagnosis (if patients), or professional background/banding (if staff), then this would elicit a ‘yes’. If descriptions provided allowed for a general sense of the participant group or setting but lacked detail, it would be marked ‘unclear’, whereas if descriptions were missing or failed to give a sense of the setting or group at all, limiting any useful conclusion from the data, then it would be marked ‘no’.

An overall quality rating was given to each paper depending on how many criteria were fulfilled within the relevant JBI tool (see Appendix 2). Papers were assessed high (green) quality if answers to all questions were ‘yes’, allowing a maximum of one ‘unclear’. Papers were downgraded to medium (amber) quality if the answer to two or more of the tool’s questions was ‘unclear’. Papers were further downgraded to poor (red) quality status if the answer to more than one of the questions was ‘no’, or if more than two were ‘unclear’.

A random selection of 10% of papers were appraised blindly in the same way by another review team member. Appraisals were compared. If ratings differed the whole team discussed points of divergence within appraisal tool answers to reach a consensus on overall quality rating. YPAG members also made several critical observations about the quality of the data in the papers they reviewed during data extraction, which were cross checked with appraisal tool criteria and overall ratings made by the review team. Key comments from these critical observations were added to the data extraction table and are represented in the discussion. Papers were not excluded based on quality, but their likely strength of evidence was considered and highlighted within results and conclusion sections.

### Data analysis and synthesis

JBI’s convergent integrated approach was used for data analysis and synthesis [28]; a narrative approach was the most appropriate given the varied nature of available evidence and heterogeneity of quantitative outcome data. All studies were tabulated in Microsoft Excel [29] according to the data extraction categories listed previously. Quantitative data from randomised controlled trials (RCTs), cross-sectional, quasi-experimental and case-control studies were converted into ‘qualitized’ data, which involved creating narrative descriptions of relevant reported outcomes. Qualitized data were pooled with the extracted data from qualitative studies and categorized according to methodological, participant or service characteristics, or thematic similarities. The data were reviewed multiple times by the research team to identify, discuss and refine trends evident in the dataset, including viewing tabulated data in multiple ways based on groupings of extraction categories and identified themes. Ultimately a set of findings embracing six themes or categories was agreed– Table 2 demonstrates the refining process.

**Table 2:** The qualitization process

Paper	Example Extraction of Raw Quantitative or Qualitative Data	Qualitized Version	Themes /Categories Identified
Casher 2003[30]	‘Finding 3. Parent/ guardian (*n* = 88) mean rating for helpful to child on the 4-point scale (1 = not helpful to 4 = extremely helpful) was 3.45 (SD =.69), and for helpful to family, 3.20 (SD =.77). When parents/guardians were asked whether or not they felt the consultation aided in their child’s recovery in the hospital, 75 (85%) indicated yes and 13 (13%) indicated no.’	‘Parents on the whole reported the service as being extremely helpful to their child. The majority felt that the psychological consultation aided their child’s recovery in the hospital.’	**Service Type** - Inpatient Paediatric Psychology, Consultation Model **Stakeholder Perceptions**– parents viewed service input as helpful and as aiding CYP recovery.
Garralda (2016)[31]	‘Discharge HoNOSCAS were obtained for a quarter of referrals and in comparison with initial scores, they documented a statistically significant decrease or improvement in psychiatric symptoms and function, with mean initial HONOSCA score 14.79 and follow up mean HONOSCA score 9.38 (p = < 0.0001) (available for 192 patients). Initial scores were higher compared to those seen (11.4) in community CAMH but level of improvement was similar (mean follow up 7.7).’	‘There was a significant improvement in clinician rated global functioning from referral to a liaison team and follow up.’	**Service Type**– Whole Hospital Multidisciplinary Team PMHL Model **CYP Health Outcomes**– clinician-rated positive impact on psychiatric functioning.
Worsley 2019[32]	‘Adolescents experienced the hospital as a safe environment, were relieved to be receiving help to reduce their suicidal thoughts or behaviours and expressed appreciation for compassionate clinicians. They emphasized the value of physical comfort, staying occupied, and information about what to expect. Reports of embarrassment and discomfort about repeated inquiries from the clinical team, and unanswered questions about what would occur during the planned inpatient psychiatric hospitalization, were common.’ Having the psychiatric technician present and being given information from the psychiatrist were highlighted as valuable aspects of the hospital stay.’	Presence of psychiatric trained staff is a valuable resource for teens admitted to the general hospital as a result of suicidal ideation. In particular, having access to someone providing observations had a compassionate approach and could answer questions information about what would happen next in terms of psychiatric input was appreciated.	**Service Type**– Inpatient Risk Support Model **Stakeholder Perceptions**– service input viewed positively by CYP, improved hospital experience. **Evidence of Integration**– psychiatric trained observation staff.

## Results

### Characteristics of included studies 

Over half (*n* = 28) of the studies were conducted in the US and one fifth (*n* = 11) in England. Others were conducted in Canada (*n* = 5), Norway (*n* = 3), France (*n* = 2), Sweden (*n* = 1), Ireland (*n* = 1), Denmark (*n* = 1) and Australia (*n* = 1).

Around half of the studies were cross-sectional in design (*n* = 27), many of these surveying staff or completing retrospective patient chart reviews. In terms of experimental studies, there was one RCT [33] and thirteen quasi-experimental studies. Of the eight studies with qualitative elements, four examined staff perspectives, three examined CYP perspectives, and two examined parent perspectives. Two mixed methods studies met the inclusion criteria, but for one of these only the qualitative element had relevant extractable data, and therefore was counted as qualitative only [34]. The other consisted of a cohort study with a qualitative element [35]; quality appraisal was completed separately for each element, as per JBI guidance.

Studies mostly investigated the impact of teams, pathways, or interventions on length of stay (*n* = 12); stakeholder experience (*n* = 14) or satisfaction (*n* = 6). Ten studies reported on mental health outcomes, and four reported physical health outcomes. Other notable outcomes assessed included the costs of hospitalization (*n* = 6) and ongoing healthcare utilization, e.g. return to ED or number of outpatient appointments (*n* = 5).

A range of acute hospital settings were represented, largely EDs (*n* = 18) and inpatient paediatric wards (*n* = 16). 14 studies examined a combination of settings within the same hospital or conducted work across multiple hospital settings. The remaining studies (*n* = 6) examined hospital-based outpatient clinics.

### Quality appraisal

Quality rating for individual studies is indicated in Appendix 2 and Table 3 by colour coding, green for high quality, amber for medium quality, and red for low quality. Where mean quality estimates for evidence related to each service type are given, these were calculated numerically by assigning 3 points for high quality, 2 for medium, and 1 for low.

**Table 3:** Key findings by service type and theme

Service Type	Studies by Service Type/Quality	Evidence of Integration	LOS	CYP Health Outcomes	Health Costs/Utilization	Stakeholder Experience
Emergency Dept. Assessment and Risk-Support; CYP admitted following SA/SH. Usual Staff: MHP, Psychiatrist or Social Worker.	Brown [45]; Casher [30]; Holder[41]; Hutcherson [52]; Jacinthe [60]; Mahajan [44]; McCabe [76]; McNicholas[40]; Nagarsekar [39]; Normand [35]; O’Donnell [77]; Parker[38]; Rotherham-Borus 1996[47]; 2000[46]; Sheridan[42]; Uspal [43]; Wharff[78]; Wolff[34]. **(n = 18) Mean Quality Rating: Medium**	Dedicated, in-person, PMHL services providing crisis interventions in the ED and supporting paediatric staff contribute to a range of improved outcomes compared to less integrated models, e.g. remote consult [30, 38, 40–47, 60, 76, 78]	PMHL service input results in shorter LOS [30, 38, 39, 41–44]	Family-focused crisis interventions in the ED and follow up care from the PMHL service may help to reduce suicide re-attempts and depression levels up to 18 months after initial ED presentation [35, 46, 47, 78].	Studies described reduced need for post-ED care following increased PMHL service input [30, 38, 42, 44, 52, 77]. One found physical restraint reduced but no change in admissions[43]; one reported reduced paediatric but increased psychiatric admissions [41]. Cost reductions were linked to shorter LOS in three studies [40, 41, 44]	Dedicated PMHL service increased paediatric staff satisfaction and perception of care efficiency for emergency CYPMH input. Desire for increased input also evident. Limited data regarding CYP and parent views of PMHL service input in ED, though most liked a post SA follow up call intervention. [34, 35, 38, 39, 43, 45, 60, 76].
Inpatient Risk-Support; CYP admitted following SA/SH. Usual Staff: Multidisciplinary.	Bowden[37]; Carter[36]; Garralda[31]; Hutcherson[52]; North[57]; Talbot[79]; Watson[51]; Weisser[66]; Worsley[32]. **(n = 9)** **Mean Quality Rating: Low**	In-person presence of PMHL service provides sense of safety and reassurance for CYP parents, and paediatric staff. Accessible mental health information and expertise important [32, 37, 51, 57, 66].	LOS not reported by any studies in this category.	CYP referred to PMHL scored better on behaviour + mental health measures than a non-referred group [36]. Significant improvements in CYP mental health and family functioning seen from referral to discharge in another service [31].	CYP who received a cognitive behavioural therapy (CBT)-based family suicide prevention intervention provided by the PMHL service were more likely to need lower levels of care than those who did not [52].	PMHL service input helpful for all stakeholders. Compassion + information about ongoing mental health care key elements for CYP. Staff support, education, clear care planning + communication important to paediatric staff [32, 36, 37, 51, 57, 66, 52,76].
Inpatient Support; CYP with LTCs or FSS. Usual Staff: Psychology.	Bowling[74], Bujoreanu[75]; Christie 1999[80]; 2003[70]; Douglas[69]; Gallagher[62]; Kullgren 2015[49]; McFadyen[81]; McGrady[48]; McKenna[61]; Piazza-Waggoner[50]; Rodrigue[59]; Vandvik[63]. **(n = 12) Mean Quality Rating: High**	Presence and availability of PMHL service allows collaborative working, quick response times to referrals, and in-person delivery of psychological therapy for CYP and families [48, 50, 59].	Shorter time to refer to PMHL predicted shorter LOS [48, 74, 75]	Pain and anxiety reduced following biofeedback intervention provided on the ward by PMHL service [61].	Referral rates to two PMHL services increased significantly over a period of 3–5 years [50, 81]. Reduced costs+ shorter LOS associated with PMHL service involvement during stem cell transplants [48]; another study measuring LOS also found associated cost reductions [75].	Paediatric and psychology staff reported high levels of need for PMHL input across all inpatient paediatric specialties, with desire expressed on both sides for an increase in mental health resources available to support this population [49, 59, 63].
Outpatient Support; CYP with LTCs or FFS. Usual Staff: Psychology.	Chambers[53]; Coburn[54]; Girling[58]; Kallesøe[33]; Lerner[55]; Rodrigue [59]; Sawchuk[73]; Sil [56]. (n = 8) Mean Quality Rating: Medium	Integrated model led to improved symptoms in SCD, diabetes + FSS [73, 55, 58], and parent satisfaction with celiac service [54].	N/A	CBT [33] /Biofeedback + CBT improved FSS [73]. See also evidence of integration (improved physical symptoms) [53, 55].	CBT intervention reduced admissions for CYP with SCD [55] but no difference observed between integrated and referral care models [56].	Improved psychology access via integrated model. High satisfaction reported by CYP, parents + referring medics alike [54, 58, 59].

Of the 27 cross sectional studies eleven were judged to be high quality, nine medium and eight low quality. These studies were comprised broadly of two types: retrospective analyses of case notes seeking to identify factors or impacts associated with the presence or absence of PMHL professionals or services, e.g., length of stay (LOS); and surveys related to staff experience or satisfaction with PMHL service. The most frequent limitation seen within this body of work was a lack of discussion around strategies to deal with confounding factors, making it particularly difficult to confidently assess the link between provision of PMHL services and outcomes in these studies. Furthermore, measures in studies asking about staff experience and satisfaction were frequently bespoke with no evidence of validation. The thirteen quasi-experimental studies were also of mixed quality. These were largely comprised of pre and post study designs exploring outcomes following implementation either of novel interventions or pathways offered by existing PMHL services. Many quasi-experimental studies were pilot designs and aimed at assessing feasibility or acceptability of methods to inform future experimental research.

There was one RCT included in the review [33], and this was assessed to be at high risk of bias due to a lack of blinding both for participants and for those delivering treatment. Cohort and case control studies (*n* = 4) were judged to be largely of medium or low quality and thus at some risk of bias, again linked to poor approach to dealing with confounders, in addition to a lack of follow up data in one case [30], and a lack of comparability across patient groups in another [36].

The one mixed methods study had a cohort study component which was judged as high quality. Its qualitative element was judged to be of low quality due to a lack of exploration of potential researcher bias or position in relation to the research [35]. The overall qualitative evidence body was small (*n* = 7). Most studies failed to report a specific research methodology and most made little or no reference to researcher reflexivity and positionality, with three omitting to state philosophical approach or link it to methodology [32, 34, 37]. This limits assessment of how the researcher’s viewpoint might have influenced data collection and analysis.

Most papers reported sex, age, and ethnicity of CYP participants, as well as primary mental health diagnosis; the demographics presented appear to be largely in keeping with the authors’ understanding of the usual characteristics of patient groups seen within PMHL services. However, there was limited discussion within the papers of local representativeness. Socioeconomic data were also largely absent, representing a weakness across the dataset. Staff participants were usually referred to by profession alone, limiting any assessment of potential gender, age or ethnicity skew impacting conclusions on staff perspectives.

### Thematic analysis 

The integrated findings of the included studies are organised according to six headings which link to the review question and aims; these are discussed below.

#### PMHL nature and function

Information was collated to allow an overview of service types described in the literature allowing a description of the variety in nature and function of PMHL services, including hospital setting, target patient group, and usual staffing. The studies described several service models; an overview of these and how they relate to one another is set out in Table 4.

ED based teams were the most frequently reported (*n* = 18). These were typically dedicated to assessing, managing, and discharging/admitting CYP with emergency mental health presentations, e.g., self-harm or suicidal ideation. The nature of the team’s role varied from telephone psychiatric consultation for ED medical staff assessing CYP [38, 39] to full multidisciplinary teams covering the whole hospital including the ED [40]. Four studies described professional pairs– most often a social worker and a psychiatrist– based in the ED [41–44].

**Table 4:** Overview of service models described in the literature

Presentation	Emergency Department	Inpatient	Outpatient	All-area
Crisis mental health presentations (Primarily suicidality, self-harm)	On- site dedicated team (psychiatry, nursing, social work) providing consultation, to staff, assessment and risk support.	Multidisciplinary mental health support offered during admission, Consultation and in-person risk-monitoring provided.	Follow up calls or appointments after ED presentation for suicide attempt, offered by team who provided initial assessment.	24/7 Multidisciplinary Team Model, dedicated on-site provision, offering a combination of work described in all columns to the left.
	Off-site, called when needed, psychiatry or nursing), providing consultation to staff and/or assessment.			
	Lone psychologist integrated into ED team providing consultation to staff.			
Psychological impact of physical symptoms	No service model for this presentation described in this setting.	Standalone cross-speciality psychology-only team offering psychological consultation to staff, assessment and/or psychological intervention to inpatients and on an outpatient basis.		
		Lone psychologist integrated into individual specialty team or clinic, offering psychological consultation to staff, assessment, and/or psychological intervention on both an inpatient and outpatient basis.		
		Psychiatry + psychology liaison team offering psychiatric and psychological consultation to staff, psychiatric and/or psychological assessment and intervention on both an inpatient and outpatient basis.		
Functional Somatic symptoms	Input from separate psychiatry and psychology teams described as part of whole hospital pathway model. Provision of psychological interventions and/or assessment of other underlying psychiatric needs and/or provision of medication.			

The extent to which these teams were integrated into the department was variable, with some located off-site and called in when needed, and others working as a part of the ED team and able to consult and advise more generally. Although no studies directly compared clinical effectiveness of different kinds of models in this category, one study found that having an on-site PMHL service was perceived to be more effective by paediatric staff [45], and several others found, compared with previously only ad-hoc or limited provision, the introduction of a dedicated PMHL service either on or off-site could improve service-use related outcomes including ED LOS [30, 38, 41, 44]; see below for further detail regarding LOS as an outcome. Three studies described models of care for ED patients requiring which included post-discharge follow-up such as check-in telephone calls [35] or therapeutic outpatient appointments [46, 47].

Another service model identified in the literature were teams that supported CYP admitted to inpatient paediatric wards. Most of these appeared to be psychologist-led with their primary function being supporting the management LTCs or FSS (*n* = 13) e.g., [48–50]. Some studies also described teams providing treatment and risk management to hospitalised CYP following self-harm or suicide attempts, sometimes pending psychiatric admission (*n* = 9). These service models were more likely be staffed by multi-disciplinary teams (MDTs) including mental health nurses [51], psychiatrists [34, 52] or in one hospital, ‘psychiatric technicians’ [32, 37].

Studies reporting PMHL work in outpatient clinics (*n* = 8) usually reported a single psychologist integrated into a physical health specialty team with a role of assessing and treating the psychological impact of LTCs, e.g. type 1 diabetes [53], coeliac disease [54], sickle-cell disease (SCD) [55, 56], and kidney disease [57]. Two studies reported a service spanning all medical specialties within the hospital [58, 59], although these appeared to be standalone services which took referrals, rather than professionals integrating into individual specialties.

Several studies additionally described teams or services which provided cover across multiple hospital settings. However, the exact scope of services in this category was poorly defined and the studies tended to explore general staff satisfaction with the service, or numbers of referrals, limiting comparisons between service models on effectiveness or outcomes. McNicholas, et al. [40], was the exception to this, providing a more in-depth comparison of three different PMHL service models operating at different hospitals in the same city. Two of the services operated what was described as a ‘medical model’, provided by psychiatrists and mental health nurses only, with input from other specialties on a case-by-case basis, offering mainly ED and inpatient support in daytime hours only. The third service, provided by an MDT, offered an on-call 24/7 service that could provide non-urgent care and outpatient follow-up in addition to ED work and inpatient support. Key findings included that LOS was statistically different between the three hospitals (p = < 0.0001), with the first hospital (medical model) mean stay at 6.67 days, the second (medical model) at 1.27 days, and the third (MDT model) at 2.13 days. The third hospital also admitted significantly fewer (*p* = 0.0001) CYP presenting to the ED compared to the other two hospitals– a difference not explicable by a difference in out of hours presentations (*p* = 0.680). The authors hypothesize that the difference is due to the presence of the 24/7 MDT at hospital three, although this does not account for the shorter LOS at hospital two which did not have an on-call service. Given the study’s short time frame (1 month), its conclusions are limited, but otherwise the study was well conducted.

#### Evidence of integration

Evidence of integration explored the extent to which teams were embedded within the acute hospital, e.g. were they located on site, did they conduct joint clinics with paediatric staff, how physically available was the team– and whether these elements were linked to stakeholder experience and patient outcomes. Table 4 provides a summary of evidence of integration in relation to the service types most frequently described in the literature and themes.

The importance of in-person team presence was a strong theme within the evidence across papers, regardless of service type or hospital area covered. Many studies described improvements in stakeholder experience and outcomes when dedicated, in-person teams or professionals were introduced to the hospital or began to provide a more closely integrated service [30, 38, 41–44, 50, 60]. From an inpatient perspective, regular in-person access to trained mental health professionals, e.g. providing 1-2-1 observations, was reassuring for CYP awaiting psychiatric transfer, and their families, enabling them to ask questions about ongoing psychiatric care [32]. For CYP requiring support with the psychological sequelae of physical symptoms, an in-person team allowed for psychological intervention to be provided during their inpatient stay, reducing symptoms such as pain and anxiety [61, 62]. For CYP attending outpatient physical health clinics, the presence of a psychologist within the clinic itself, working together with paediatric staff during initial assessment and subsequently, was associated with improved physical health outcomes and parent satisfaction [53–55]. There is more detail on these studies in the section on CYP health outcomes and stakeholder experiences.

Five studies conducted surveys to investigate staff satisfaction with interprofessional working between paediatric departments and CAMH services and views on service integration [15, 63–66]. Although these surveys were largely superficial and did not consider concepts related to integration in-depth, the overall conclusions indicate that closer integration between mental health staff and paediatric departments was seen as desirable by paediatric staff, but overall, there was some dissatisfaction with current levels of collaboration or interprofessional working, and an expression of an increased need for access to the PMHL service. The study reported by Woodgate and Garralda [15] was the only study to examine specific liaison activities thought to promote closer integration with acute care such as a hospital base (59%), engaging in joint outpatient appointments (52%) or conducting joint rounds (19%); these features also appear in the wider integrated care literature as potential indicators linked to improvements in patient outcomes [67, 68]. Other studies identified that paediatric staff particularly valued provision of education around mental health care by the PMHL service and forums for case discussion or staff support [57, 69–71] and clear communication and care planning [34, 57].

 Two papers evaluated whole-hospital integrated care pathways for CYP with diagnoses of FSS [72, 73]. Kullgeren et al. [72] found that 55 inpatients receiving a new multidisciplinary care pathway had significantly shorter LOS (n2 = 0.06) and fewer subspecialty consults (odds ratio 0.24 [95% CI 0.10–0.54]) plus reduced median costs compared to a control group of 53 CYP not enlisted onto this pathway, saving an estimated $51,433 per inpatient episode. Participants in the pathway group discharged from the paediatric ED had lower costs relative to comparator groups in the study. Sawchuck et al. [73] found a statistically significant reduction of non-epileptic seizure frequency in CYP treated on the pathway (p = < 0.001). Length of time in remission status was 13.4 in the control cohort compared to 3 months in pathway cohort. In terms of healthcare utilization there was an 86% reduction (p = < 0.001) in ED visits pre and post diagnosis. There was also a 74% reduction in non-epileptic seizure episodes requiring an ambulance callout pre-diagnosis vs. post treatment initiation (p = < 0.001). PMHL services are explicitly mentioned within both pathways and, although their specific input or influence on outcomes is not explored statistically or otherwise, they are a reminder of the complexities involved in the vertical integration required to provide care to CYP with both physical and psychological symptoms, particularly when physical symptoms are suspected to at least have some psychogenic qualities. In both of these cases, pathways were developed jointly and agreed between the acute and mental health teams, demonstrating the importance of close holistic working practices with clearly defined roles for groups of CYP requiring physical and psychological care, and illustrating a wider point about the function of PMHL services as having a crucial role in determining a more psychologically minded approach to provision of care acute hospital settings.

#### Length of stay

Length of stay (LOS) was the most reported service-level outcome measure and referred to whether receiving care from the PMHL service could be said to have an impact either on time spent in the ED, or as an inpatient. Details of the 12 papers which measured LOS, either in ED or inpatient stays, can be found in Appendix 2.

Many of these papers (*n* = 8) explored the impact of providing a PMHL service in an ED setting. Six studies evaluated the impact of PMHL service input within the ED on time spent within the ED itself [30, 39, 41–44]; finding a mean reduction in LOS of 1.7 h, *p*-values ranging from 0.0001 to 0.055 and a mean quality rating score of ‘medium’. McNicholas [40], notes a statistically significant difference for inpatient LOS (p = < 0.001) between the hospital with a 24/7 PMHL service covering the whole hospital (mean LOS 2.13 days) compared to for patients admitted at two other hospitals offering a less comprehensive, office hours only services (hospital 1 mean length 6.67 and hospital 2 mean length 1.27). Although its LOS was not the shortest of the three, the hospital with the 24/7 MDT admitted significantly fewer (*p* = 0.001) patients presenting to the ED (40.6%) compared to hospital 1 (83.3%) and 2 (93.3%), highlighting the need for other contextual data to fully understand the significance of LOS as an outcome in this population.

Three studies analysed data on the time taken for physical health teams to refer to inpatient PMHL services after admission [48, 74, 75]. These all concluded that a shorter inpatient LOS had a statistically significant association (*p* = 0.0001 to *p* = 0.001) with a shorter time from admission or referral to PMHL, to initial consultation with a PMHL professional. However, none of these studies provided much (if any) detail about the consultation or subsequent treatment CYP received from the PMHL service, beyond being either psychologist [48], or psychiatrist-/psychologist-provided [74, 75], and that it focused on psychological issues linked with physical illness. Kullgren (2020) [72] analysed data from two groups of CYP receiving inpatient treatment for FSS; one who had received a new multidisciplinary team care pathway (*n* = 32), the other who had not (*n* = 19). As detailed in ‘Evidence of Integration’ section, their conclusion was that the new pathway reduced LOS; however, in addition to limited detail regarding PMHL service involvement in the pathway, group sizes were small, and the control group was from a different time point, introducing the possibility of uncontrolled time-specific confounders. Overall, however, studies measuring LOS were judged to be of high quality and of numbers which give a good indication that PMHL service input can significantly reduce LOS in the ED and can support shorter inpatient admissions for CYP requiring physical health or functional symptom treatments.

#### CYP health outcomes

CYP health outcomes refers to whether either a referral to the PMHL service or specific interventions provided by the PMHL service could be associated with either physical or mental health outcomes, e.g., suicidality, pain or overall functioning. There were few studies which measured any CYP health outcomes at all (*n* = 11). Three of these were judged to be of low quality, due to incomplete follow up [78], lack of appropriate measures or steps taken to account for confounding factors [73], and lack of treatment blinding [33]. Just three studies measured both physical and mental health outcomes; a coaching intervention to improve diabetic control [53], CBT for CYP with SCD [56], biofeedback for pain and anxiety [61], and Acceptance and Commitment Therapy for overall physical and psychological functioning in FSS [33], all of medium or high quality. There were also four studies providing some indication that PMHL and targeted interventions in the ED may have some impact on suicidality and depression at follow-up [35, 46, 47, 78] though the varied measures and timeframes employed in the studies, undermined further by a lack of reported statistics concerning long-term follow-up or derived from comparison with suitable control groups, limits this observation. Multidisciplinary pathway approaches for CYP with FSS were investigated in two studies [72, 73], which concluded that a holistic approach which included PMHL could reduce unnecessary investigations, improve communications, and hasten recovery in this group of CYP. Results of these studies are explored in greater detail in the ‘Evidence of Integration’ section. However, it must also be noted that the effect of PMHL service input specifically was not measured or isolated within these pathway studies, and therefore it is not possible to determine the extent to which the PMHL service was implicated in the positive outcomes reported. Only one study [15] specifically evaluated the impact of referral to a PMHL service, concluding significantly positive effects on CYP MH and family functioning, on the basis of improvements seen in HoNOSCA score from referral to discharge from the PMHL team (p = < 0.0001). Although the study was appropriately powered (*n* = 192), and the HoNOSCA is as a valid measure of psychiatric functioning [82], it must be noted that is completed by clinicians at the point of discharge from services, potentially introducing bias linked to service pressure to demonstrate improvements in order to retain funding. Despite variation in study quality and measures used, studies assessing health outcomes do consistently suggest that integration of mental health professionals who can provide psychological interventions in acute paediatric settings can positively impact on physical and mental health outcomes.

#### Cost and healthcare utilization

Healthcare cost and utilization covered any service-level outcomes aside from LOS; for example, ongoing admissions, returns to ED, or costs associated with healthcare use. Six studies measured costs associated with hospital stay [40, 41, 44, 48, 72, 75]. These mainly concluded that the reduction in healthcare costs observed were a consequence of reduced LOS. Kullgren *et* al (2020) linked costs reductions to reduced investigations during treatment FSS [72], as previously discussed. Healthcare utilization, e.g. number of return ED visits, inpatient admissions, or outpatient use, was reported as an outcome associated with PMHL service input in six studies [30, 38, 55, 56, 72, 73]. Results in these studies conflicted, with some finding an association between PMHL service use and a reduction in subsequent outpatient appointments or ED use, e.g., Parker [38] and others, e.g., Lerner [54] finding no significant difference between groups receiving integrated or non-integrated PMHL care models. In one study [30], an integrated care intervention in the ED actually appeared to result in an increase in hospital admissions in the short term compared to a control group and a brief nursing intervention group. However, the integrated intervention was associated with a reduction in longer-term admission rates and outpatient appointments, indicating that longitudinal data may be key in determining the true impact of PMHL input on service-level outcomes.

Given the reasonable strength of the evidence and number of studies reporting on it, PMHL service provision is likely associated with reduced admission costs by virtue of a reduction in length of stay. However, there is not enough evidence to assume any such association with healthcare utilization, nor to conclude that reduction in LOS would necessarily lead to reduced overall healthcare costs.

#### Stakeholder perceptions and experiences of PMHL

Twenty-nine papers reported data related to stakeholder perceptions of PMHL service provision. Most of this work consisted of staff surveys (*n* = 17) or interviews (*n* = 4). Parent views were also ascertained via survey (*n* = 6) or interviews (*n* = 2). Relevant CYP views were ascertained via interview in three papers.

Studies reporting on staff perceptions indicate overwhelmingly that paediatric staff value the integration of mental health trained staff into the acute setting. Paediatric staff appear to value being able to easily access mental health professionals for advice and support around the psychological aspects of healthcare, and many papers indicated that staff would like more education and clinical input from PMHL services [15, 60, 65, 66, 69, 70, 80]. There were clear indications that paediatric staff found it important and helpful for PMHL service staff to be easily accessible within the acute paediatric setting, e.g [45, 69, 83].,, with frustration if this was not the case, as in e.g. Jacinthe [60]. Overall it seemed that paediatric staff would like closer integration, e.g., employing mental health nurses on paediatric wards [51], feeling it would be helpful in supporting better quality care for CYP, and that what was on offer was not always the most helpful approach. Indeed, as in the study reported by Christie in 2003 [70] dissatisfaction may well arise when there is a perceived disconnect between what is offered by PMHL service staff to ward staff and what is actually needed, or where input feels unclear or sporadic. One study [45] indicated that both satisfaction levels and effectiveness were positively influenced where teams appeared to be integrated into the MDT rather than contracted in, and had their own established space in the hospital. There was an overarching theme that establishing a PMHL service or increasing PMHL staff presence generally improved awareness and knowledge of psychological presentations for paediatric staff, which in turn demonstrated to paediatric staff, the need to have mental health trained staff at hand. As one staff member nicely summarised it in the paper by Nisell [65] *‘the more they are here*,* the more obvious the need for them.’;* this theme is illustrated nicely by the group of four papers reporting doubling or even trebling of referrals during time periods ranging from 1 to 5 years following the introduction or augmentation of a PMHL service in the hospital [50, 71, 81, 84].

Several studies obtained the views of PMHL service staff directly, however there was a lack of detailed qualitative work, with many of studies simply asking respondents to rate their own work, service, or perception of cooperation with paediatric colleagues on a Likert type scale [15, 43, 49, 52, 63, 66]. In the few studies which elicited more detail, concerns were raised relating to the difficulty in providing psychological care in an acute setting, for example safety issues related to managing aggressive behaviours in the paediatric ED [76], limited access to suitable rooms for mental health assessment [34], or the chaotic nature of the acute ward as a barrier to providing regular psychological intervention [61].

Data related to parent experience are comparatively sparse (*n* = 7). Bowden [37], focussing on the experiences of parents with a child admitted to the paediatric hospital while waiting for psychiatric admission, found that they valued the specialist support and information that mental health professionals in the service provided; Carter [36] found 75/85 of parents whose CYP were referred to the PMHL service believed the team input had aided in their child’s recovery. From an outpatient perspective, the study by Coburn [54] suggested that having the opportunity to explore the psychological impact of an LTC without needing to go to a separate provider or appointment was a priority for families, and that the input from the team had felt supportive, echoing similar findings by Girling [58]. Talbot and Malas [79] investigated the effectiveness of a video introducing the PMHL service to parents in reducing the stigma around accessing help for CYP with mental health difficulties, with results which appeared somewhat inconclusive, not least because of the lack of any kind of control group. They reported a limited shift in parental attitudes overall, but a statistically significant increase in confidence that a PMHL service could help their child (*p* = 0.017), and that care would be confidential (*p* = 0.001). Questions examining beliefs about over-medication (*p* = 0.018) and potential negative long-term effects of medication for behavioural difficulties on long-term development (*p* = 0.048) also saw a positive shift. These findings chime with detail from the study reported by Christie [70], suggesting the PMHL service has a broader role to play in reducing stigma around provision of mental health care in the acute setting. Given the poor quality and lack of depth of many of the studies exploring parent perspective however, the dataset can overall only really provide a general indicator of parent experiences of PMHL services and limits the strength of conclusions regarding specific elements of the work which are helpful for families.

Just three studies reported on CYP experiences of using PMHL services. Normand, et al. [35] sought to evaluate the acceptability of a telephone follow-up intervention for CYP who had been seen in the ED by the PMHL service following suicide attempts: the intervention had a mixed-reception from participants with some finding it to be helpful and supportive (and would have appreciated more phone calls) while others disliked being reminded of their suicidal episode. Worsley, et al. [32] interviewed 27 CYP admitted to paediatric wards following self-harm or a suicide attempt and who were waiting to be admitted to psychiatric units. CYP reported valuing the presence of mental health trained staff during their stay, particularly for the quality of the interactions experienced, and because their presence ensured they were able to ask questions about their ongoing psychiatric care and receive appropriate information. The third, study, a survey-based study [58], collected a very limited amount of data regarding CYP’s experiences of a paediatric psychology service, reporting that although the majority (*n* = 32, 97%) of CYP would recommend the service, some reported feeling uncomfortable with a family approach to treatment, while others felt that therapy was repetitive or patronising. Given the paucity of this body of evidence, with all studies exploring CYP views rated as low quality, it is of limited usefulness in establishing what might be important to CYP using PMHL services.

## Discussion and conclusion

This mixed-methods systematic review has synthesised an international body of evidence on the range of impacts of the provision of PMHL services in acute hospital settings. Studies were reviewed that report on a range of service configurations demonstrating the broad scope of what can be understood as PMHL and the potential benefits of locating mental health professionals in acute paediatric hospital settings. This includes: the ED, for assessment and management of acute mental health presentations; on paediatric wards to provide psychological support to CYP admitted awaiting psychiatric transfer or requiring physical treatments; and as part of outpatient clinics to provide psychological input alongside medical colleagues. The review also locates PMHL teams as a part of a broader acute system supporting the treatment of CYP with complex presentations where the cause of physical symptoms is unclear, such as functional somatic syndromes.

Many included papers reported cross sectional, questionnaire studies seeking the opinions of broader acute hospital staff groups regarding their experience of referring to or utilising PMHL services of a variety of configurations. Although the methodological quality of these studies was limited, their volume did strengthen the credibility of the thematic findings. Overall, it was clear that acute hospital staff value the presence and input of PMHL services and mental health professionals; that frustration arose when PMHL were perceived as inaccessible or inadequately integrated into the medical specialties they served; and that increased PMHL service presence, input, and education was desired by physical health staff. PMHL service presence also served to increase the knowledge and awareness of the psychological aspects of physical illness for paediatric staff and demonstrate the need for mental health input in medical settings. This fits with findings from research related to adult mental health liaison, such as Wood and Wand [18]. However, the superficial nature of most of the studies exploring staff satisfaction and experience limits more in-depth conclusions regarding the nuances of how identified elements of PMHL service practices function to support positive outcomes for CYP. There was a limited amount of research that collected data from PMHL services across different hospitals in the same city or country, however these tended to ask closed Likert type questions about satisfaction with collaboration or explore to what extent respondents felt their work to be important, rather than providing any real detail about what services were on offer or associated outcomes. Detailed exploration of PMHL staff views and experiences was largely absent, with the limited data providing some indication of the challenges faced by staff providing psychological or mental health support in acute paediatric settings. Further qualitative work would bring valuable insights to experience of both physical health and PMHL staff working in these settings with CYP requiring physical and mental health care.

Increasing the presence and integration of PMHL services and mental health professionals into settings traditionally geared towards the treatment of physical symptoms only, allowing for increased recognition of both the psychological antecedents and sequelae of physical illness, makes sense in the context of calls for integrated health services as one way of working towards the ideal of parity of esteem between mental and physical health care [85], as seen for example in UK mental health policy directives since 2011 [86]. Data from included studies does indicate that that relying on externally located CYP mental health services may be associated with poorer outcomes and reduced satisfaction from acute hospital staff and that co-location of dedicated, closely integrated PMHL services provide increased opportunities to find solutions in what are often complex or challenging healthcare presentations; something that has been identified as a key element of effectiveness in recent research looking at mental health services for CYP experiencing common mental health problems such as depression [87]. The need to wait for assessment from an external team is potentially harmful for CYP with mental health needs presenting to the acute hospital; this model was associated with slightly lower perceived effectiveness than in-house or ‘hybrid model’ PMHL service by one US study [45], longer LOS by another [44], and higher rates of admission and ED return visits in two others [38, 42]. The absence of on-site PMHL service support is also reported as a potential safety issue by other sources, e.g. England’s Care Quality Commission [88]. National and international survey work would help improve understanding of the current level of PMHL service provision and integration. In England, for example, in contrast with the ‘Core 24’ requirements for adult services [89], there is no requirement for PMHL services to exist or to meet any particular standards. There is also very limited indication of the nature of, or variation in, PMHL service provision beyond an understanding that it is lacking in comparison with adult services [16].

In terms of directly linking PMHL service provision to health and service level outcomes, it is difficult to draw firm conclusions about causality due to the limited methodological quality of the included studies, an over-reliance on cross-sectional designs, and a lack of information concerning the detail of interventions offered by PMHL services particularly where studies were not focusing on a specific intervention. Nonetheless, around a quarter of the included studies were quasi-experimental and most of these were judged as being of medium to high quality. From these studies, there is a strong indication that PMHL service provision is linked to reduced LOS, with a more moderate indication that it reduces hospitalization costs and healthcare utilisation, and may improve health outcomes such as depression, suicidality, and anxiety for CYP requiring mental and physical healthcare in the acute setting. Further experimental research is necessary to strengthen these conclusions and establish causality– indeed, with only one RCT in the entire data set, there is a clear gap here for high quality experimental research to investigate the efficacy of mental health interventions provided by PMHL services. However, the challenges of conducting true experimental research around provision of often complex interventions in acute hospital settings with a group of CYP who are likely to be distressed must be acknowledged, and so it is arguable that in spite of the methodological flaws, available evidence suggests there is potential for mental health professionals in the acute setting to provide services and interventions that can directly improve both health and service level outcomes. Given the current financial struggles facing many health services, as well as the increase in presentations of CYP with mental health needs to acute hospital settings, these findings may be of interest to policy makers and commissioners. For example, a relatively cost-effective strategy may be to provide additional training to mental health professionals already located in acute settings in providing targeted interventions aimed at reducing the likelihood of a need for a return to the acute setting, e.g. telephone follow up, brief targeted therapy sessions.

What is less apparent from this review is evidence surrounding the provision of risk-focused support for CYP admitted to paediatric wards following self-harm or suicide attempts or awaiting a psychiatric bed. Most studies reporting on inpatient paediatric settings were focused on the provision of psychological interventions for CYP admitted for physical health reasons primarily, with just six papers describing risk support models [32, 34, 37, 51, 52, 79], only one of which [52] reported effectiveness data. This lack of evidence is concerning in the context of recent reports which indicate a large increase in numbers of CYP requiring care in paediatric inpatient wards, and associated concerns regarding patient and staff safety [2]. A recent systematic review of evidence related to admissions to paediatric wards for mental health reasons also found no evidence for effective intervention [5], underlining the gap in the evidence and the likely need for development of PMHL services in this setting. Given the likelihood that a more closely integrated physical and mental health care workforce is likely to improve outcomes, and the clear desire for increased access to practical, in-person mental health support by paediatric staff, a potential avenue for future research might include the impact of directly employing mental health nurses and mental health trained support workers as part of paediatric ward teams.

Although the indications from the small number of qualitative papers included in this review which interviewed CYP and their parents were that the presence of mental health professionals and PMHL services linked to acute settings were valuable, and improved the care experience, the poor methodological quality of these papers– particularly the lack of theoretical or reflexive integrity– severely limits these findings. Additionally, as they were all single centre studies, their transferability is limited. Furthermore, the focus of these qualitative papers tended to be on experience generally, without any explicit exploration of stakeholder views on areas for service development or research. To direct future research and intervention appropriately, the experiences of both CYP and their parents accessing PMHL services need to be considered, and the health and service outcomes which are important to them need to be established.

One key point pertaining to this arose during an in-depth discussion of a random selection of papers with two members of the study YPAG who checked data extraction and commented on how they could relate the findings to their own experiences of using English CAMHS, including PMHL services. One YPAG member expressed their concern that many of these studies frame reduction of LOS as a positive outcome, when in fact from a patient perspective this is not always the case, as sometimes getting better takes time, rather than pushing for an early discharge, which may inadvertently lead to re-referrals and re-admissions. The study reported by O’Donnell, et al. [77], was highlighted as an example of this; they found some CYP were discharged merely because they or their parent/carer was tired of waiting for transfer to a psychiatric unit, rather than because they were ready or felt safe to be discharged home. Interestingly, the only study which described a comprehensive multidisciplinary PMHL model in line with the adult Core 24 standards, reported lower admission rates and costs per admission, but longer length of stay in comparison to a medical model psychiatry service with no on-call provision [40]. An additional consideration raised by the YPAG members in relation to LOS in the studies they reviewed was the lack of information concerning the legal frameworks in use; indeed, there was no mention of any legal frameworks in any of the included studies. In a similar vein, YPAG members highlighted that in the single study reporting on the use of restraint practices [42], the study’s conclusions did not differentiate between chemical and physical restraint, something that could be of great importance to service users. This raised the question of firstly, why there was no differentiation, and secondly why such an important outcome was not considered in other studies. Finally, on reading a draft of the completed review, one YPAG member provided an additional comment that an overall lack of representation of patient views is a major weakness within the literature.

Although this, like many aspects of evidence from this review, requires significant further research to be able to provide any level of certainty with regards to the specifics, in broad brush terms the body of evidence strongly that PMHL services are essential across all acute hospital settings, their presence ensuring timely access to mental health assessment, risk support, and psychological therapies for to CYP requiring physical and mental health care. Evidence related to integration was, overall, of a medium quality, and– despite some flaws - supports the ecological validity of the assertion that in-person work involving a joint approach between PMHL services and paediatric staff, as well as specific targeted interventions appropriate to hospital setting and presentation, is likely to yield better patient and service level outcomes, as well as stakeholder satisfaction, compared to on-call or consultation only models where PMHL service staff do not work directly alongside their paediatric counterparts. It is therefore imperative that they be commissioned and funded sufficiently if we are to work towards improving outcomes and reduce ongoing healthcare costs for this group of CYP.

### Strengths and limitations

A major strength of this review is its mixed methods approach which allowed for the inclusion of both qualitative and quantitative studies, making it the first systematic review of its kind on the topic. This approach enabled identification of a range of outcomes, including those related both to acceptability and effectiveness of services or interventions. The variety of PMHL models, health and service level outcomes, and stakeholder perspectives represented in the included papers together provide a comprehensive and holistic view of PMHL services in a range of acute paediatric settings and provide multiple avenues for further research or practice innovation. A further strength of this review is its adherence to the JBI methodology for integrative systematic reviews. This ensured a thorough and systematic approach at all stages, enhancing the reliability of the conclusions. The inclusion of the YPAG members in the review process was an additional strength, providing lived-experience insight. The review was limited somewhat by the lack of duplicate data extraction, potentially introducing an element of selection bias; however, efforts were made to mitigate this as far as possible within the limitations of a PhD project, i.e. data from 20% of the included papers were extracted by two reviewers and assessed for consistency.

The resultant heterogeneity in interventions and outcomes, given that PMHL services may be defined and implemented differently across settings and mental health presentations, posed a challenge to an integrative synthesis. Additionally, search terminology and exclusion criteria meant that studies retrieved were likely to only be from countries where the term ‘mental health liaison’ is recognised and where publications were available in English. It is not known whether this term is universal or whether the practice of PMHL exists or has a different label elsewhere. It is also possible that there is a lack of published studies in countries where stigma around mental health treatment is significant or in low-income countries where they may be insufficient funding, motivation, or infrastructure for research. In addition, the body of literature is from countries with different health care systems (e.g. insurance and non-insurance-based healthcare services), where different diagnostic criteria and labelling and financial incentives may affect the comparability of some outcomes. This limits extrapolation of findings beyond the largely high-income countries represented in the included studies. In terms of the included studies, the main limitation is the reliance on cross-sectional designs, and the variable methodological quality of the included studies, both of which severely limit the strength of conclusions linking PMHL service provision to relevant health and service level outcomes. The included qualitative research was particularly poor methodologically, highlighting a need for further high-quality studies exploring stakeholder perspectives, particularly those of CYP whose viewpoint was poorly represented.

### Recommendations for practice

A general, JBI grade B (‘weak’) recommendation for practice is for PMHL services to be available in all acute hospitals and be as closely integrated as possible with acute care provision.

Available evidence suggests that key elements of PMHL service provision particularly valuable in the acute setting are:

- Round-the-clock access to mental health professionals able to provide CYP-focused psychiatric assessment, consultation and in-person risk support in the ED and on paediatric wards. Specific examples of effective integrated practice would include:

- Mental health practitioners, with robust support from child psychiatry, available on site 24/7 to provide holistic assessment of any CYP mental health presentation or suspected mental health presentation in the ED or on a paediatric ward.
- Co-located mental health practitioners, and mental health-trained support workers available as part of the paediatric staff for practical risk-support input such as providing 1-2-1 support for CYP at risk of harm to self or others; advising around appropriate and safe restraint practices if needed; implementing mental health care plans suitable for CYP and setting.
- Attendance of mental health professionals within paediatric MDT meetings as routine, both to be able to provide consultation and initiate direct intervention when indicated.
- Consultation from child psychiatry available when needed, particularly to advise regarding use of mental health act and on complex cases where symptom cause is unclear, risk to self or others is high, or psychiatric medications are indicated.
- Provision of dedicated space within the hospital to allow the PMHL service a permanent and accessible presence within the hospital and appropriate private space for on-site assessment and treatment of CYP and families.

b) Psychological consultation and long-term intervention available both in paediatric wards and outpatient settings. Mental health professionals need to be able to provide psychological therapies suitable for addressing the challenges associated with experiencing physical ill health, e.g. Acceptance and Commitment Therapy, Biofeedback.
c) Mental health professionals who fulfil each of the above roles should be employed as part of the multidisciplinary team within each physical health specialty. Ideally, they need to be supported by a dedicated on-site multidisciplinary mental health team including, at minimum, child psychiatry, clinical psychology, mental health practitioners and mental health support workers.

### Recommendations for research

High quality, qualitative research is needed to explore in-depth staff, parent, and CYP experiences of PMHL. This should prioritise ascertaining the acceptability of services to CYP and obtaining insights into service provision challenges and facilitators from PMHL staff, as well as ascertaining the specific support needs of paediatric staff.

Work is required to identify priority research outcomes, particularly those of importance to CYP and families.

High quality experimental, prospective research is needed to strengthen findings regarding the impact of PMHL teams on health and service level outcomes, particularly for CYP.

Up to date survey and cross-sectional work to establish the current landscape of PMHL provision is needed, including comparative studies to help support evaluation of differences between models.

Further research identifying and evaluating models of care focusing on inpatient paediatric risk support and outpatient clinics.

## Electronic supplementary material

Below is the link to the electronic supplementary material.

Supplementary Material 1

Supplementary Material 2

Supplementary Material 3

## Data Availability

No datasets were generated or analysed during the current study.
