# Supplementary material for: A mixed methods systematic review of the impact of paediatric mental health liaison services on children and young people’s mental and physical health, stakeholder experience, and service-level outcomes
Source: Eur Child Adolesc Psychiatry. 2025 Jul 15;34(12):3749–67. doi: 10.1007/s00787-025-02815-5 (PMC12743064; doi:10.1007/s00787-025-02815-5)
Supplement: Supplementary file 1 — Supplementary Material 1 [file 787_2025_2815_MOESM1_ESM.docx]

| Appendix 1. Combinations of Search Terms used in each Database. | | | | | | |
| --- | --- | --- | --- | --- | --- | --- |
| Database | **MeSH? or similar option used.** | **Wildcard** | **Truncation** | **Paediatric** | **Mental Health** | **Liaison** |
| Ovid Medline | Yes. | ? | * or $ | child* or young or p?ediatric* or youth or juv* or teen* or adolesc* | "mental health" or psychiatr* or psychol* or mind-body | liaison or consult* or integrat* or multidisciplinary or MDT or crisis or emergency or "rapid response" |
| PsycArticles (on Ovid plat) | Apply related words + apply equivalent subjects (will also search plurals) | ? | * or $ | " | " | " |
| PsycBooks | Yes. | ? | * or $ | " | " | " |
| PsycInfo | Yes. | ? | * or $ | " | " | " |
| AMED | Yes. | ? | * or $ | " | " | " |
| Embase | Yes. | ? | * or $ | " | " | " |
| Health and Psychosocial Instruments | No. | ? | * or $ | " | " | " |
| HMIC | Yes. | ? | * or $ | " | " | " |
| EBM Reviews (all combined) | No. | ? | * or $ | " | " | " |
| Social Policy and Practice | No. | ? | * or $ | " | " | " |
| CINAHL | Yes. | ? | * or $ | " | " | " |
| PubMed | Yes - does it automatically. | ? | * | child or children or young or p?ediatric* or youth or juvenile or teen or teenage* or adolesc* AND "mental health" or psychiatr* or psychol* or mind-body AND liaison or consult* or integrat* or multidisciplinary or MDT or crisis or emergency or "rapid response" | | |
| PROSPERO | No. |  |  | ‘child mental health’ or ‘paediatric liaison’ or ‘mental health liaison’ or ‘psychiatric liaison’ or ‘integrated care’ or ‘psychological medicine’ or ‘emergency mental health’ or ‘paediatric mental health’ or ‘rapid response’ (searched as separate phrases). | | |
| ClinicalTrials.Gov | Yes - automatically searches synonyms |  |  |  | mental health | liaison |
|  |  |  |  | child | mental health |  |
