# Supplementary material for: A mixed methods systematic review of the impact of paediatric mental health liaison services on children and young people’s mental and physical health, stakeholder experience, and service-level outcomes
Source: Eur Child Adolesc Psychiatry. 2025 Jul 15;34(12):3749–67. doi: 10.1007/s00787-025-02815-5 (PMC12743064; doi:10.1007/s00787-025-02815-5)
Supplement: Supplementary file 2 — Supplementary Material 2 [file 787_2025_2815_MOESM2_ESM.docx]

Appendix 2. Overview of key information for each study colour coded with quality appraisal rating (red = low, amber = medium, green = high).

| **Author** | **Year** | **Country** | **Design** | **Setting** | **Population** | **Relevant Outcomes** | **Study overview including details of service model or intervention** | **Key Findings** |
| --- | --- | --- | --- | --- | --- | --- | --- | --- |
| Black | 1999 | UK | Cross- Sec | Paediatric Hospital | CYP | Referral rates | 192 referrals from paediatric departments to child mental health services were analysed before and after establishment of a regular MDT paediatric liaison meeting including paediatricians, social workers, paediatric nurses, a psychologist, psychiatrist, and mental health nurse. | After PMHL input was formalised in the hospital, there was a significant increase in child mental health referrals, indicating increased awareness of psychiatric conditions as well as a need for a PMHL service at this hospital. |
| Bowden | 2022 | US | Qual | Inpatient | Parents | Experience | 14 parents/carers with children admitted to one paediatric hospital following a mental health crisis were interviewed. The hospital is described as having a mental health consultation service, with psychiatric technicians (‘bachelor’s prepared hospital employees with additional training in child development and behaviour management’) for 1-2-1 observations. | Presence of trained mental health professionals in acute setting is important to parents/carers whose child is admitted to a paediatric ward whilst waiting for psychiatric admission. Information about psychiatric admission and reassurance was valued highly but not always available when needed. |
| Bowling | 2022 | US | Cohort | Paediatric Hospital | CYP | LOS | 302 referrals from paediatric departments to a PMHL team were analysed to examine whether time from admission to referral to the PMHL team impacted length of stay. The service is described as a 'parallel specialty service, separate and non-integrated hospital department available to all patients on referral…composed of psychologists, psychiatrists and psychiatry trainees who focused on psychological factors that are affected by medical issues'. No definition of 'consultation'. | Shorter referral time to PMHL service was associated with overall shorter length of stay. |
| Brown | 2009 | US | Cross-Sec | PED | Staff | Response time | Acute staff from 34 hospitals with PMHL services surveyed about response time for psychiatric evaluation, perception of PMHL effectiveness + other key elements of PMHL service provision. 85.8% of respondents described using either in-house or contracted nonmedical psychiatric specialists such as social workers, licensed counsellors, or psychologists to provide MH care to paediatric patients. | Perceived effectiveness of PMHL was greater in acute hospitals which had in-house paediatric mental health consultation arrangements and dedicated physical space at the hospital. |
| Bujoreanu | 2015 | US | Cross-Sec | Inpatient | CYP | LOS | 308 consultations at one PMHL service were analysed for associations with length of stay and hospital costs. Team consisted of 3 child and adolescent psychiatrists, 1.5 child psychologists, 1.0 child and adolescent psychiatry residents, 1.5 predoctoral psychology interns, and 2.0 postdoctoral psychology fellows. Team function described as providing consultations to general medical and surgical inpatient units, and remaining involved in the patient care until discharge, but no specifics about this described. | The authors estimated that after adjusting for confounders a 10% decrease in referral time to their service was associated with a 7.9% shorter length of stay (95% CI, 6.4-9.5, p=<0.001), and a 7.9% decrease in total hospital charges. |
| Burket | 1993 | US | Cross-Sec | Paediatric Hospital | Staff | Expectation of child psychiatry | 73 paediatricians from one hospital paediatric department surveyed about use of PMHL including expectations of the service, provided by psychiatrists only. Consultation is defined as process in which the psychiatrist evaluates the patient, forms an opinion, and makes recommendations to referring paediatrician. Liaison work described as psychiatrist becoming more involved with the paediatric treatment team on a continuing basis to contribute to patient care when appropriate. | Paediatricians rated accessibility and timeliness as very important when accessing paediatric mental health liaison consultation. Follow-up, liaison, and specific recommendations rated as 'important'. Knowledge of wards and participation in rounds rated as 'somewhat important.' |
| Carter | 2003 | US | Case-Control | Inpatient | Staff+ Parents | Satisfaction; Goal attainment.  Behaviour issues. | 104 referrals to a PMHL service matched with 104 non-referrals to assess impact on nurse and parent-rated behavioural and adjustment difficulties + goal attainment and satisfaction ratings from referring physicians, parents/guardians, and psychiatrist. Team described as a clinical and training/teaching unit providing consultations to paediatric inpatients at a children's hospital and consisting of a the service director (a doctoral paediatric psychologist), a child psychiatrist, two paediatric psychology doctoral fellows, a child psychiatry fellow, and a predoctoral child psychology intern. | Referrals scored higher than non-referrals on all parent-reported behaviour problems outside of the hospital (p=<0.05), and most nursing reported in-hospital behaviour problems (0.5), plus depression, sense of inadequacy and social stress. Parents felt PMHL service helpful in aiding recovery. Physicians rated PMHL team highly in terms of patient goal attainment and helpfulness. |
| Casher | 2022 | US | Cohort | PED | CYP | LOS + outpatient episodes. | 71 cases referred to a new integrated mental health care model for patients presenting to the PED. Doctoral-level psychology resident embedded with the main ED reviewed the patient's medical course, conducted brief clinical assessment, delivered brief psychotherapy/psychoeducation + coordinated with follow-up providers. Interventions includes relaxation training, motivational interviewing, parenting training + behavioural safety planning. Control groups saw a psychiatric nurse external to the ED team for triage and brief safety assessment, or if high acuity admitted to an on-site 16 bed ward 24/7 staffed by psychiatrists and nurses providing brief psychotherapy, medication management, safety planning, and care navigation (including transfer to inpatient psychiatric ward if needed). | Integrated psychology intervention in the ED reduced length of stay and outpatient episodes at 30 and 90 days after discharge. |
| Chambers | 2022 | US | Quasi-Exp | Outpatient | CYP | HbA1c | 21 boys and 19 girls received a coordinated care plan that included the expertise and availability of a certified diabetes care and education specialist (CDCES) “coach” to integrate medical care with psychology while reinforcing messaging of physical, emotional, and behavioural health. Following a visit to a paediatric endocrinologist, the CDCES met with participants and families to outline goals and establish a communication strategy to support frequent contact until the next in-person clinical visit at 4 months. Motivational interviewing was used to emphasise small, achievable, behaviour changes, monitored by the coach. Each participant also met with a psychologist who worked in the endocrine division and had expertise regarding T1D in childhood. | Integration of a psychologist into the diabetes team reduced HbA1c levels in the short and longer (8 month) term and saw an improvement in diabetes related quality of life reported by both CYP and parents. Missed school days were also reduced following intervention. |
| Christie | 1999 | UK | Cross-Sec | Paediatric Hospital | Staff | Satisfaction | 20 paediatric nursing staff across haematology and oncology were surveyed following the introduction of a permanent clinical psychologist role. The psychologist provided 3 sessions per week across two inpatient wards, as well as an outpatient/day patient ward. Time for weekly psychosocial meetings, clinical supervision and administration were included in this, with face-to-face patient contact time equalling 2 sessions or 1 day. | Nursing staff had limited knowledge of the psychology offer for their ward, and over half were dissatisfied with the service. They felt that they needed increased input and availability from the psychology service. |
| Christie | 2003 | UK | Cross-Sec | Paediatric Hospital | Staff | Experience | 25 paediatric nurses and 9 psychological liaison staff were surveyed about the interventions provided by the psychological service at one acute hospital. Two paediatric wards, an adolescent medical unit, and a teenage cancer unit, were supported by the psychological liaison service, made up of clinical psychologists, psychotherapists, and psychiatrists providing patient initial/ cognitive assessments; individual therapy; attending ward rounds/handovers; counselling for CYP and families; staff consultation; education and training of other staff groups; staff support; counselling supervision; group work; investigation of treatment effectiveness; systemic consultation to family. | Nursing staff on a paediatric ward viewed psychological input favourably and felt that assessment and therapy were priority areas. MDT approach identified as an important service feature and nurses felt that having psychology input had a positive impact on patient care. Ease of access and availability highlighted as important issue, as well as difficulty doing this kind of work in this environment. |
| Coburn | 2020 | US | Cross-Sec | Outpatient | CYP + Parents | Experience | 48 families completed a post-clinic survey regarding satisfaction with a psychological consultation which had been provided as part of multidisciplinary celiac disease clinic appointment. The clinic appointment consisted of 30-minute appointments with each member of the team (gastroenterologist, dietitian, gluten-free education specialist, psychologist, and neurologist). | Most parents reported being very satisfied with a psychology consultation given as part of a multidisciplinary coeliac clinic, with comments made about the importance of MDT approach with all appointments/ team members in one place on one day. |
| Douglas | 2015 | UK | Qual | Paediatric Hospital | Staff | Experience | 6 staff (paediatricians, paediatric nurses, AHPS) interviewed about use of a weekly psychosocial forum in a paediatric hospital setting. This is described as a weekly multidisciplinary meeting established by clinical psychologists in medical settings providing psychological consultation to multidisciplinary team members, with a focus on the psychosocial factors influencing current patient care. The meeting can also be used for coordination of referrals, supervision of other team members, teaching opportunities, and the arrangement of research collaborations. | Staff in a paediatric hospital viewed the forum as 'essential' to the service and were mostly very satisfied with it. Staff valued the opportunity to discuss cases and consult the psychologist for advice and feedback as well as teaching. Focus on holistic approach and opportunity to consider psychological issues important. Staff felt more psychology hours would be useful. |
| Gallagher | 2014 | US | Cross-Sec | Inpatient | Staff | Perceptions | 19 clinicians from a PMHL service were surveyed following training/providing a biofeedback intervention. These included 3 child psychiatrists, a nurse practitioner,4 child psychiatry residents, 2 post-doctoral psychology fellows and 6 pre-doctoral psychology interns. Biofeedback was provided to inpatients referred to the team for anxiety or pain management, and consisted of physiological monitoring whilst relaxation exercises were guided by the clinician, to enable patient observation of changes to physical state post-relaxation, and increased symptom mastery. | Staff trained in a biofeedback intervention felt more confident following training/implementation that provision of the intervention could increase patient and parent receptiveness of psychiatric intervention in a paediatric hospital setting. |
| Garralda | 2016 | UK | Quasi-Exp | Inpatient | CYP | HoNOSCA Score | 192 CYP referred to a multidisciplinary paediatric liaison team. Clinical outcomes (specifically the clinician rated HONOSCA score which considered degree of improvement in psychiatric and social status and family function) were compared to national community CAMH data. Team consisted of one full time child and adolescent consultant psychiatrist and one part time family therapist, providing care to young people on inpatient paediatric wards at a specialist acute hospital. | There was a significant improvement in clinician rated global functioning from referral to a liaison team and follow up, with some level of improvement reported for most child psychiatric and family problems, child psychosocial functioning, and family functioning. |
| Girling | 2016 | UK | Qual | Paediatric Hospital | CYP +Parents | Satisfaction | 45 CYP and families interviewed about use of a clinical psychology service for CYP under the care of a hospital consultant. The team is embedded in the paediatric and adolescent service with referrals often received following MDT meetings, from medical specialties including weight management, oncology, chronic fatigue, pain disorders, urology, endocrinology and diabetes. The team provide specialist inpatient, day and outpatient care to support CYP and families in managing issues raised by their illness and its treatment and build emotional well-being. | Both young people and parents overwhelmingly said they would recommend the paediatric clinical psychology service at the hospital. Work was described as helpful and effective with positive results. |
| Holder | 2017 | US | Quasi-Exp | PED | CYP | LOS | 1048 CYP treated pre-PMHL team introduction compared to 1613 after. Pre-program, the PED had limited access to evaluations by staff with MH expertise. ED social worker was available 20 hours per day and psychiatrist 4 hours per day. Post-program, 9 social workers were given enhanced training and psychiatry supervision. Psychiatry time increased by 8 hours/day. Daily team meetings + case reviews were held with the social workers where the psychiatrist reviewed cases, provided education + liaison services to ED physicians + clinically evaluate and treat complex cases. | Introduction of a dedicated liaison team in PED led to a significant reduction in length of stay, and fewer admissions to paediatric units (but an increase in transfer to psychiatric hospitals). |
| Hutcherson | 2021 | US | Cross-Sec | PED + Inpatient | CYP+ Staff | Disposition | 218 CYP presenting to ED or on medical wards following suicidal ideation or attempts received a cognitive behavioural suicide prevention family intervention called SAFETY-A. The intervention aims to build coping skills for CYP and families, increase motivation for post-discharge treatment, and improve connection to outpatient treatment after discharge from the ED. This was provided by psychiatrists, social workers, psychologists, and licensed professional counsellors working in the hospital who had received training in the intervention. | Patients who completed all components of the SAFETY-A intervention provided in the ED were more likely to need lower levels of care than those who did not. There was a mixed response from clinicians about the extent to which the use of SAFETY-A influenced their decision making. (See also Rotheram- Borus as intervention previously tested there). |
| Jacinthe | 2010 | CAN | Cross-Sec | PED | Staff | Experience | 87 ED staff were surveyed about a crisis intervention programme that had been introduced to help manage psychiatric patients presenting at the ED. Crisis Intervention Workers (CIWs), masters-educated in social work or counselling, provide mental health assessments with an emphasis on risk assessment and treatment recommendations, plus consultation + staff education. Onsite coverage weekdays 07:00 to 24:00 hours, and limited daytime and evening coverage is available during the weekend. Psychiatrists and psychiatry residents are on-call as needed. | ED staff generally satisfied with the implementation of a crisis intervention program in the ED but wanted increased availability of CIWS. Staff comments suggested that they valued the expertise of the clinicians in the department, and that they felt this improved management/assessment of YP in the department and linking them to local resources post-discharge. |
| Kallesøe | 2021 | DEN | RCT | Outpatient | CYP | Physical Health (SF-36 Score) | 44 CYP with functional somatic syndrome (FSS) randomised to receive the AHEAD intervention in addition to usual care. The manualised group‐based cognitive therapy was given in groups of seven to eight patients with nine modules (i.e., 27 h in total) over a period of 3 months and one follow‐up meeting (3 h) 3 months after the last module. This was provided by 2 psychiatrists and one psychologist, all with Acceptance and Commitment Therapy training and specific knowledge on FSS. Each group had two therapists, that is, one psychiatrist and one psychologist. | Over time the intervention group had statistically significant reductions in somatization and maladaptive illness perceptions. A clinical but not statistical difference in physical health scores and patient overall impression of change for the intervention group was seen compared to the treatment group. No significant difference between interventions primary defined endpoint. |
| Kullgren | 2015 | US | Cross-Sec | Inpatient | Staff | Experience | 127 psychologists or trainees surveyed about the practice and training of consultation liaison. This was defined as those who 'work with the paediatric inpatient medical team to identify, diagnose and evaluate problems, provide evidence-based cognitive behavioural interventions, and arrange for community-based follow up.' | Most liaison psychologists indicated they were satisfied in their work, and more than half believed they had adequate resources and support most or all the time. Over half commented on the lack of availability of outpatient resources to refer patients to. |
| Kullgren | 2020 | US | Quasi-Exp | PED + Inpatient | CYP | LOS | 71 CYP with FSS treated under a new care pathway integrating physical health care and mental health care via thorough biopsychosocial assessment, early mental health involvement, and caution with invasive, low-yield interventions and diagnostic testing. It includes provisions for verbal and written information for families, including standardized educational handouts. Paediatric psychology and psychiatric consultation services are embedded in the wider hospital and form part of the pathway, in addition to social work in psychiatry consultations for complex cases. | Inpatients receiving care under the new pathway had a significantly shorter LOS, fewer subspecialty consults, and reduced median costs than those who were not put onto the pathway. The pathway group discharged from the PED had lower costs relative to the comparator groups. |
| Lerner | 2018 | FRA | Quasi-Exp | Outpatient | CYP + Parents | Healthcare use. Cognitive recall. | 155 children with sickle-cell disease (SCD) from 129 families were compared across 3 different centres with different service models (C1-C3). C1 all SCD follow-up consultations were conducted jointly by a paediatrician and a clinical psychologist, working in close collaboration with the hospital paediatric team. C2 all SCD follow-up consultations were conducted by a paediatrician alone with referrals to a psychologist if needed + close links with local psycho-medical paediatric care centre for specialized referrals. C3, SCD follow-up consultations were organized by a paediatrician alone. | No significant difference between centres on number of hospitalization episodes or number of days hospitalized. Statistically significant (p=0.05) difference between centres for cognitive recall scores, with C1 having the greatest number of patients achieving a predetermined threshold, followed by C2. |
| Lygre | 2020 | NOR | Cross-Sec | Paediatric Hospital | Parents | Experience | Survey of 148 parents of patients aged 6-12 with at least one hospital episode in a 2-year period and with three or more primary referrals, including at least one to CAMH and one to a paediatric department. The hospital is described as providing specialist healthcare for children but there is no information provided about the different departments accessed by the survey respondents. | Perception of shorter waiting times and effective collaboration between hospital departments was associated with an experience of feeling well cared for by the hospital. |
| Majahan | 2007 | US | Cross-Sec | PED | CYP | LOS | Chart analysis of 561 patients referred to a new PMHL team in ED, 561 prior to team implementation and 500 for non-MH visits. Team was staffed (24/7) by a psychiatric social worker and a child psychiatrist. Regardless of payer, child psychiatrist supervised social worker to evaluate all CYP MH presentations. Prior to team implementation referrals were made to a variety of external MH providers. | LOS in ED for CYP requiring MH assessment reduced from 259.49 to 216.39 minutes, mean difference of 43.10 minutes (p=0.0001) reduced following the implementation of the team. Reduction in LOS was estimated to result in savings of $10,651.00 |
| McCabe | 2019 | CAN | Qual | PED | Staff | Experience | 23 paediatric and mental health staff, service managers, and other staff stakeholders were interviewed about the process of integrating a team of mental health nurses into a PED. | Team implementation coincided with increased referrals, and paediatric staff felt overwhelmed/underprepared for associated challenges. MH staff felt uncertain in treating medical aspects of MH emergencies. Managing aggressive or violent behaviour in general PED setting posed a safety issue. |
| McFadyen | 1991 | UK | Cross-Sec | Paediatric Hospital | CYP | Referral rates | 159 referrals to a PMHL service were reviewed from different time-points in a three-year period. Improvements to the service occurred prior to this but are not specified. Psychiatrists were involved in assessment and treatment more often than non-medical members of the team, but the composition of the wider team is not described. The service provided family therapy primarily, plus individual psychotherapy, behaviour therapy and parental counselling. | Referrals to the service increased significantly over the 3 years studied, whereas referrals for self-injury remained the same. In Year 1 Total referrals were 116, Year 2 104, Year 3 124, with those termed 'liaison', (i.e., excluding self-injury) 46, 43 and 70 respectively (p=<0.005). |
| McGrady | 2018 | CAN | Cross-Sec | Paediatric Hospital | CYP | Costs + LOS | Chart review of 75 CYP who underwent stem cell transplant and had one or more visits from a clinical psychologist during admission. Visits were defined as individual or family therapy provided by a licensed clinical psychologist with specialized training in paediatric psychology. Visits conducted for assessment purposes only (e.g., neuropsychological evaluation) were excluded. | Longer time to consultation (TTC) significantly predicted longer LOS (p<.001). Each day increase in TTC was associated with 0.7% increase in length of the initial SCT hospitalization when effects of covariates were held constant. |
| McKenna | 2015 | US | Quasi-Exp | Paediatric Hospital | CYP | Pain | 66 CYP admitted medically + referred to PMHL for pain and/or anxiety management + received Biofeedback intervention. Every patient received a formal psychiatric assessment by PMHL clinicians (psychology/psychiatry attending, psychiatry nurse practitioner, psychiatry/psychology fellow, or psychology intern) before the intervention, provided by a PMHL clinician trained in the intervention (see Gallagher 2014 for description). Participants had on average 1.6 x 21-minute-long sessions. | Pain scores decreased following sessions across all groups – significantly more in the group with both pain and anxiety than in the groups with just pain or just anxiety (p=0.057). Significant improvements in emotional state seen in all groups post-intervention (p=0.0001). |
| McNicholas | 2021 | IRE | Cross-Sec | PED | CYP | LOS | 59 CYP presenting across three EDS + referred for urgent review by PMHL services in Nov 2016. Hospitals 1 and 2 had psychiatry + MH nurses covering ED + wards only in office hours only, 5 (H1) or 7 (H2) days per week. Hospital 3 provided 24/7 MDT coverage for all departments doing emergency, inpatient and outpatient work. | Statistically significant difference noted between length of stay for patients admitted at the different hospitals (p=<0.001). Hospital 1 (mean length 6.67 hours), Hospital 2 (mean length 1.27 hours) Hospital 3 (mean length 2.13 hours). |
| Nagarsekar | 2021 | AUS | Quasi-Exp | PED | CYP | LOS, carer satisfaction. | Of 219 CYP presenting to ED for MH over a 3-month period, 50 (23%) used the new KALM pathway, and 169 (77%) had treatment as usual. The KALM pathway involved use of a standardised risk assessment tool (HEADSS) by a paediatric or emergency medicine registrar who then consulted an on-call psychiatrist to create a plan, and referred to a MH clinician if this was advised or if the case was complex. Treatment as usual involved assessment by a specialist MH clinician (external to dept). | Patients on the KALM pathway had an average LOS of 58 minutes less than those on TAU, statistically significant at p=0.055. Patients on the KALM pathway were also significantly less likely to breach the department’s 4 hour wait target. No overall difference in carer satisfaction between the two pathways but this was significant when comparing subset of those who breached when asked whether carer felt their child was stressed by the process/if they were overall happy. |
| Nisell | 2007 | SWE | Qual | Paediatric Hospital | Staff | Experience | 100 nurses, 11 program directors representing a range of medical specialties, and 5 executives were interviewed about co-operation between child psychiatry and paediatrics before and after a move to integrate psychiatry into different somatic specialties. | Staff indicated that mental health care was frequently needed for their patients, and they would benefit from more education and training, as well as closer cooperation with mental health professionals in the hospital. Presence of MH professionals was linked to increased recognition of MH needs in patients. |
| Normand | 2018 | FRA | Qual (?MM) | PED | CYP | Suicide reattempts at 1 year | 173 CYP seen at the PED following a suicide attempt were provided with a one-year telephone follow up intervention. Data was collected about repeat suicide attempts and participants were interviewed about their experience. The intervention was provided by the PMHL team which consisted of one psychiatrist, one psychiatric trainee, one psychologist and two nurses. | At one-year follow-up, 23/173 CYP had reattempted suicide (13.3%). Of the 23 patients, 14 had reattempted at least once during the one-year period (15%). There were a total of 18 reattempts, 8 of which occurred between the six-months and the 1-year phone contact. The intervention generally received a positive response from CYP and parents. |
| North | 1998 | UK | Cross-Sec | Paediatric Hospital | Staff + CYP | Satisfaction | 18 staff (mainly paediatricians, junior doctors, nurses) surveyed regarding PMHL service satisfaction. PMHL is described as not integrated with clinical psychology, but MDT including consultant psychiatrist, junior medical staff, and psychiatric social workers. Access to a child psychiatry day unit run by another consultant with a full nursing and social work team is also mentioned. No dedicated allocation of time or resources was made for the paediatric liaison aspect of either consultant’s work | Paediatric staff reported high levels of satisfaction with the PMHL service and valued accessibility, open communication, opportunity for case consultation, and clear guidance. Staff support e.g., after a death, was also seen as a key function of the PMHL service. |
| O'Donnell | 2021 | US | Case-Con | PED | CYP | Disposition | 79 CYP presenting to ED for MH and recommended for inpatient psychiatric hospitalization, compared with 316 control group patients matched for age, sex and race to establish differences between patients discharged from the medical hospital whilst waiting for a psychiatric bed, and those who went on to psychiatric admission. All CYP had an initial psychiatric evaluation by a social worker specialised in mental health care or a child psychiatrist but limited therapeutic resources thereafter. The hospital had 15 inpatient paediatric psychiatric beds with access to more at nearby hospitals if required. | CYP discharged home without needing psychiatric admission were more likely to have received a formal consult from a psychiatrist whilst in PED, (p =.001) + have received haloperidol for agitation (p=0.022). Most common reason for discharge 'no inpatient bed availability, guardian tired of waiting' (69.1%), 58.8% ‘patient improved/stable’. 33.% reported a guardian had requested discharge, with 27.8% saying they felt safe to do so. |
| Parker | 2003 | CAN | Cross-Sec | PED | Staff + CYP | Admission rates + LOS | 168 CYP seen in ED by PMHL prior to the implementation of a rapid response model compared with 172 CYP seen in first year of implementation. The model consisted of one child psychiatrist providing emergency consultations either in person or over the phone in the ED; this also included a diversion system whereby lower-risk cases could be booked directly into outpatient appointments later in the week to avoid an ED wait. The psychiatrist also provided education for providers using the service. | The presence of the rapid response model was positively associated with reductions in; ED admissions (p=<0.05), non-ED admissions (p=<0.05); ED contacts without admission (p=<0.05) and average LOS (p=<0.05), with presence or absence affecting all aside from ED contacts without admission. Paediatric staff felt efficiency was improved and were generally satisfied. |
| Perera | 2008 | UK | Cross-Sec | Paediatric Hospital | Staff | Experience | 17 staff from a paediatric hospital MDT (including nurses, a consultant, play therapist) surveyed regarding the implementation of a PMHL service. PMHL service included a consultant psychiatrist (6 sessions), specialist nurse (5 sessions) and a psychotherapist (1 session). There is no detail provided about the scope of the team or interventions provided. This is an audit and therefore of limited methodological quality. | PMHL perceived as a valuable service; 89% felt they offered useful advice. Respondents reported improvement in MH care, faster response + better staff support. 2/3 respondents + all consultants, thought more sessions were necessary. It was noted that having an ‘‘on-site’’ PMHL team makes referral and joint working easier, particularly in terms of parent acceptability and accessibility. Referrals to PMHL doubled over a two-year period. |
| Piazza-Waggoner | 2013 | US | Cross-Sec | Paediatric Hospital | CYP | Referral rates | 1,749 referrals reviewed following implementation of an inpatient paediatric psychology service 3 clinical psychologists hired + trainees on rotation, providing consults to all medical, surgical, and intensive care units in the hospital. The service uses a family systems model, with consults available to patient, caregiver, or both. Care is described as collaborative with the paediatric team including understanding how professionals impact the patient/caregiver presentation. Input is provided until issue is resolved or discharge. Prior to this psychology input was ad-hoc with no dedicated staff. | Demand for the service almost doubled over the 5 years studied, with 257 referrals received in year 1 and 508 in year 5. Numbers of teams requesting consults also increased over time. The top three referring services across most years were neurology, gastroenterology, and general paediatrics. Highest representation of caregiver referrals was from intensive care. |
| Rodrigue | 1995 | US | Cross-Sec | Paediatric Hospital | Staff | Satisfaction | 43 staff surveyed about their use of the Paediatric Psychology Service with a modified version of Olson and co-workers' (1988) questionnaire. Faculty physicians accounted for the largest percentage of respondents (41%), followed by medical residents and fellows (23%), nurses (11%), social workers (11%), dentistry faculty (4%), and others (e.g., psychiatrists, psychologists; 10%). Sixty-one (43%) respondents identified themselves as working with primarily paediatric patients, while 82 (57%) respondents indicated working primarily with adult medical patients. Female respondents accounted for 46% of returned questionnaires. Paediatric Psychology Service described as staffed by five full-time licensed psychologists, including a paediatric neuropsychologist, plus predoctoral graduate students/interns and postdoctoral fellows. Service offering ‘a wide range of clinical child, paediatric psychology, neuropsychology, medical psychology, and general mental health services’. Consultation requests to see patients in the health centre (e.g., inpatient paediatrics, medicine, psychiatry) are typically handled on the same day, while outpatients are seen on an appointment basis. | Of those who had requested psychology consultations within the last 12 months (n=190), the overall return rate was 75%. Respondents had requested approximately two consultations per month, on average, over the past 6 months and thought slightly less than one-third (30.1%) of their patients would benefit from a psychology consultation. On a scale of 1 (not at all satisfied) to 5 (very satisfied), Mean satisfaction with overall services was 4.0, with 75% endorsing category 4 or above. Response time mean 3.7, 65% at 4+. Usefulness of feedback and recommendations 4.0, 79% at 4+. Written feedback 3.5, 76% at 4+. Verbal feedback 3.5, 50%. At 4+ Intervention effectiveness 3.6, 59% at 4+ Outpatient follow up 3.3, 41% at 4+. |
| Rotheram- Borus | 1996 | US | Quasi-Exp | PED | CYP + Parents | Suicidality | 140 female adolescents presenting consecutively to the PED after a suicide attempt. 75 participants and their families received standard ED care; subsequently 65 attempters and their families received a specialized ED program including an in-ED therapy session by a ‘crisis therapist’, additional staff training (for both psychiatry and paediatric ED staff), a soap opera video shown to parent, and a referral to SNAP follow up outpatient therapy (six-session structured family-oriented program, therapist training not specified). 75 randomised to standard care: assessment in ED by a psychiatrist and referral to SNAP follow up outpatient therapy. | CYP in the specialized ED program reported lower levels of depression on the BDI and less suicidal ideation on the HASS scale when assessed after the emergency room intervention (post-discharge assessment) than did those who received standard care. Similarly, mothers in the specialized program reported lower levels of depression on the BDI and less overall psychopathology on the BSI. CYP receiving the specialized program were significantly more likely than CYP in the standard care condition to return to the clinic for any outpatient treatment following their discharge from PED. |
| Rotheram-Borus | 2000 | US | Quasi-Exp | PED | CYP + Parents | Suicidality | 150 CYP admitted to ED following a suicide attempt. 65 randomised to specialised care including an in-ED therapy session by a ‘crisis therapist’, additional staff training (for both psychiatry and paediatric ED staff), a soap opera video shown to parent, and a referral to SNAP follow up outpatient therapy (six-session structured family-oriented program, therapist training not specified). 75 randomised to standard care: assessment in ED by a psychiatrist and referral to SNAP follow up outpatient therapy. | At 18-month FU there were 11 suicide re-attempts in the standard care group and 6 in the specialised care group. Time to suicidal re-ideation varied based on number of sessions attended, suggesting attendance at 7+ sessions was protective for CYP with low-moderate symptoms. Depression scores were lower in the specialised care group at follow up. |
| Sawchuck | 2020 | CAN | Cross-Sec | Paediatric Hospital | CYP | Remission  Rates | 42 CYP with a diagnosis of psychogenic non-epileptic seizures (PNES) treated on a new clinical care pathway including clinical psychology assessment, standardised CBT, biofeedback, and in-hospital psychiatry or specialized somatisation outpatient clinic as needed. Comparison group was 29 CYP included in an earlier cohort to validate the pathway. | Both cohorts had similar remission rates - development cohort at 59% full/21% partial, validation cohort 63% full/12% partial. Average monthly frequently of PNES events significantly reduced from 44 at diagnosis to 1.7 at discharge (p=<0-.001). |
| Sheridan | 2015 | US | Quasi-Exp | PED | CYP | LOS, use of restraint, disposition,. | 212 CYP presenting to ED for MH, 83 before and 129 after the implementation of a PMHL team. The team consisted of a psychiatrist + social worker providing ‘direct therapy and interventions’ in the ED, although no detail is provided. The social worker also provided 1-month outpatient follow-up for CYP whilst they transitioned to appropriate outpatient providers. | Median PED LOS was 24.0 h preintervention and 20.9 h postintervention This was a decrease of 27% and was determined to be statistically significant (95% CI 0–46%; p = 0.05). No statistically significant difference in restraint use pre and post intervention period. Patients admitted/transferred to an inpatient psychiatric facility was 42% in the preintervention year, decreased to 24% in the postintervention year (p=<0.01) |
| Sil | 2020 | US | Quasi-Exp | Paediatric Hospital | CYP | Healthcare utilization | 101 CYP with SCD referred to paediatric psychology outpatient psychotherapy clinic for pain management, comparing those who completed CBT treatment with those who did not. Sessions were between 45–60 minutes. Treatment plans were tailored to meet the individualized needs of patient and family, accounting for age, distance from clinic, complexity of medical follow-up, comorbidities affecting chronic pain, and patient preferences. Treatment provided by 4 licensed clinical psychologists and 4 psychology postdoctoral fellows trained in evidence-based CBT interventions for chronic pain. | CYP who terminated CBT early had increased admission rates over time relative to comparisons; those who established care had a faster reduction in admissions over time relative to comparisons. 18 CYP (31.6% of the treatment group) completed patient-reported outcomes reporting significant decreases in pain intensity, functional disability, and improved coping efficacy from pre- to post-treatment. |
| Talbot | 2019 | US | Quasi-Exp | Paediatric Hospital | Parents | Perception of PMHL service | 30 parents/carers of CYP who had used the paediatric hospital for a variety of mental and physical health difficulties were shown a brief educational video and asked to complete a stigma questionnaire before and after. The video introduced PMHL members/services + address specific aspects of MH stigma. The PMHL service was inpatient only, providing assessment and intervention. Team members were a psychiatrist, a paediatric nurse practitioner, a social worker, and a child and adolescent psychiatry fellow. There is also a separate psychology service who the PMHL joint work with when appropriate. | Statistically significant increase in confidence that PMHL could be helpful (p = .017) but no significant differences in caregiver attitude questions. Concerns regarding MH confidentiality decreased significantly following viewing the video (p = .001) and stigma improved relating to overmedication (p = .018) + long-term negative effects of psychotropic medication (p=.048). |
| Uspal | 2016 | US | Cross-Sec | PED | CYP+ Staff | LOS, restraint, admission, staff satisfaction. | 738 CYP who visited the ED for MH before and 902 after introduction of a dedicated MH team based in ED 24/7 providing evaluations, coordinating care, providing behavioural interventions + brief psychoeducation to CYP+families. Team is mental health evaluator (nurse with at least 2 years of psychiatric nursing experience/master’s degree social worker) + paediatric MH specialist (a ‘bachelor’s degree–level provider’), role to support behaviour management in ED, discharge planning + crisis management training for CYP + families. ED physical environment also adjusted to enhance safety. | A significant decrease in mean ED LOS was observed postintervention, from 332 mins (95% CI = 309–353 mins) to 244 mins (95% CI = 233–254 mins). No changes in admission rates or 72-hour return rates observed. There were significant decreases in security physical intervention events (2.0% vs. 0.4%, p = 0.004) and use of physical restraints (1.7% vs. 0.1%, p < 0.001). Staff satisfaction significantly increased post-intervention concerning the ED’s process for evaluating and managing patients with MH concerns, family satisfaction, making appropriate disposition decisions, and process safety. |
| Vandvik | 1994 | NOR | Cross-Sec | Paediatric Hospitals | Staff | Experience | 53 child psychiatric outpatient units and 25 paediatric departments surveyed about perceptions of collaboration between psychiatry + paediatrics in hospital settings. At time of survey there were 5 regional paediatric departments and 20 paediatric hospital departments. The first child psychiatric department opened in the Paediatric Clinic at the National Hospital in 1950, with a consultation/ liaison service with paediatrics formed in 1958. At time of survey this was a team of eight (child psychiatrists, clinical psychologists + clinical social workers) working full time in the National Hospital. | Response rate over 90% both psychiatry (responded to by both psychologists + psychiatrists) + paediatrics. No significant difference in collaboration satisfaction. All wanted increased PMHL services. Many wanted more education on PMHL work + more evaluation/therapy of patients with cancer, transplantations, psychosomatic diseases + organic brain syndromes |
| Watson | 2006 | UK | Cross-Sec | Paediatric Settings in a Trust | Staff | Experience | 90 nurses working in paediatric wards across one Trust surveyed about their attitudes + concerns regarding nursing CYP with MH needs. PMHL run by psychiatry and psychology departments described in the hospital, but author identifies need for improved nursing liaison. Liaison nursing is described as focusing 'on the interpersonal relationship between nurses and patients rather than on diagnosis and treatment of illness’ and providing 'support and advice to nursing colleagues in the general paediatric setting who have 24-hour responsibility for the wellbeing and safety of this client group'. | Most nurses did not feel confident in nursing this group of CYP + identified a need for training. Many wanted MH nurses included in paediatric ward staffing. Many felt unsupported by MH professionals. On-call support was rated as a beneficial aspect of PMHL, along with education on conditions/management, individual support for CYP+families, staff support, and care planning. |
| Weisser | 2019 | NOR | Cross-Sec | Inpatient | Staff | Satisfaction | 14 staff at CYP psychiatric wards providing a PMHL + 34 staff at CYP somatic wards that use a PMHL service, surveyed about organization of + satisfaction with the content, quality + availability of PMHL services across Norway. Respondents appeared to be medics but this was not made explicit, nor the staffing composition of PMHL services in question. | Most respondents reported above average satisfaction with PMHL services but thought that scope should be increased. Service availability was poor out of office hours. Most somatic doctors wanted an outpatient PMHL clinic to follow up patients with trauma, recently diagnosed cancer, suicidal behaviour + other conditions. |
| Wharff | 2012 | US | Quasi-exp | PED | CYP | Repeat suicide attempts at 3 months | 100 CYP presenting to ED with suicidal ideation before and 150 CYP after introduction of a pilot of a family-based crisis intervention. CBT and family systems approach to one session provided in the ED by a social worker, who explores CYP + family narrative of what has happened + what would be necessary for CYP to return home safely. Post-intervention social worker consults with supervising psychiatrist to review case. Discharge only when agreed by CYP, patient, family, psychiatrist, + social worker. | No patients in the intervention group reported incidence of attempted or completed suicide during the three-month follow-up period. CYP who presented to ED during study period had significantly fewer psychiatric admissions than members of the matched sample who presented during the comparison period. |
| Wolff | 2023 | US | Qual | PED | Staff | Experience | 15 staff involved in care of CYP presenting to PED for MH, including PMHL (psychiatry, psychology, social work) and paediatric staff (paediatricians, registered nurses), interviewed about: strengths and contributors to success in keeping patients safe while awaiting inpatient psychiatric care, obstacles to quality care, bottlenecks in patient flow, and potential points of intervention by which to alleviate burden and strain on patients, families, hospital staff, and resources. | Strong communication, collaboration + leadership across teams highlighted as key to supporting safe/efficient patient journey. Difficulties were inappropriate space, patient acuity/volume, staff availability, and long wait times for this patient group. All felt increased MH input + funding was needed. |
| Woodgate | 2006 | UK | Cross-Sec | Paediatric Hospital | Staff | Satisfaction | 19 paediatricians (63% RR) + 27 specialist multidisciplinary CAMHS providers (RR 73%) surveyed to explore the nature and extent of PMHL services provided in London. Paediatric liaison defined as joint working across professional boundaries; care delivered by more than one specialty or profession. Consultation: what happens when a specialist, either working individually or as part of a team, gives an opinion on a client or patient to another professional seeking advice - often without seeing the patient. Opinions were asked of both groups of professionals about the perceived quality of services they were providing (CAMHS) or utilising (paediatrics). | Only 1/3 of respondents felt the MH needs of CYP were being met. Virtually all paediatricians wanted to develop more links with/receive more input from MH professionals. Virtually all felt paediatric liaison was an important area to target paediatric and CAMH resources. |
| Worsley | 2019 | US | Qual | PED+Inpatient | CYP | Experience | 27 CYP aged 9 to 21 years hospitalized for self-injury or suicide attempt, medically cleared, and awaiting transfer to an inpatient psychiatric unit were interviewed to understand CYP perspectives during boarding hospitalizations to gain insight into helpful practices and targets for improvement'. PMHL input as follows: Evaluation by ED physician, social worker, and psychiatrist to determine the severity of physical and mental health concerns following presentation to ED with self-harm or suicide attempt. If medical treatment required or psychiatric bed required but not available, patient is admitted to a medical inpatient or observation unit and cared for by a general medical team, mental health consultation service, paediatric nursing staff, and continuous 1:1 safety observer. Rounds are most often conducted without patient or family present, and individual clinicians inform patients and families of updates after rounds. Most safety observers are psychiatric technicians (i.e., bachelor’s-prepared hospital employees with training in mental health or child development).' | Adolescents experienced the hospital as a safe environment, were relieved to be receiving help to reduce suicidal thoughts or behaviour and expressed appreciation for compassionate clinicians. They emphasized the value of physical comfort, staying occupied, and information about what to expect. Reports of embarrassment and discomfort about repeated inquiries from the clinical team, and unanswered questions about what would occur during the planned inpatient psychiatric hospitalization, were common.' Having the psychiatric technician present and being given information from the psychiatrist were highlighted as valuable aspects of the hospital stay. |
