# Supplementary material for: A mixed methods systematic review of the impact of paediatric mental health liaison services on children and young people’s mental and physical health, stakeholder experience, and service-level outcomes
Source: Eur Child Adolesc Psychiatry. 2025 Jul 15;34(12):3749–67. doi: 10.1007/s00787-025-02815-5 (PMC12743064; doi:10.1007/s00787-025-02815-5)
Supplement: Supplementary file 3 — Supplementary Material 3 [file 787_2025_2815_MOESM3_ESM.docx]

**Appendix 2 – Papers with Length of Stay as an Outcome**

| **Paper** | **Intervention or Service Description** | **Findings related to Length of Stay.** |
| --- | --- | --- |
| Bowling 2022  (US) | 'Parallel specialty service, separate and non-integrated hospital department available to all patients on referral.' 'Composed of psychologists, psychiatrists and psychiatry trainees focused on psychological factors that are affected by medical issues'. 'Consultation' is not defined. | Shorter time to consultation with the service was associated with shorter length of stay (p=0.001). Half of the differences between hospitalization lengths explainable by time to initial consult with the service. A cumulatively negative effect of delaying referral to the service is concluded. |
| Bujoreanu 2015 (US) | 3.0 full-time equivalent child and adolescent psychiatrists, 1.5 child psychologists, 1 child and adolescent psychiatry resident, 1.5 predoctoral psychology interns + 2 postdoctoral psychology fellows providing consultations to general medical and surgical inpatient units. Attending psychiatrist/psychologist performed consultations with a trainee + remained involved in care until discharge. No further info re: team role. | A positive correlation of 0.34 was found between referral time and observed-to-expected length of stay (P = .0001). After adjusting for psychiatric functioning, physical illness severity and psychiatric disposition a 10% decrease in referral time to the service was associated with a 7.9% shorter length of stay (95% CI, 6.4-9.5, p=<0.001). |
| Casher  2022  (US) | Integrated care intervention (doctoral psychology resident embedded in main ED team) **VS** triage + brief safety assessment by psychiatric nurse (not part of main team) **VS** Psychiatric Emergency Service admission. On-site 16 bed ward 24/7 staffed by psychiatrists and nurses providing brief psychotherapy, medication management, safety planning, psychiatric transfer if required. | A statistically significant difference in median LOS was observed between groups, with the integrated care intervention group averaging 3 hours **VS** 4 in the brief nursing intervention group **VS** 8 for the psychiatric emergency service group (p=<0.01). |
| Holder 2017  (US) | 9 ED social workers given enhanced training + ongoing supervision from psychiatrist. Psychiatrist time increased:8 hours/day (+ 4 hours consultation time previously provided). Psychiatrist held daily team meetings/case reviews with social workers, provided education/liaison services to ED physicians + evaluated/treated complex cases. | PED LOS decreased significantly from 14.7 to 12.1 hours (P < 0.001) following the implementation of the program. |
| Kullgren 2020  (US) | Somatic symptom disorder pathway inc. biopsychosocial assessment, early MH involvement, caution with invasive interventions + diagnostic testing, info for families, discharge plan/resources, provider comms guidance. MH services not directly available to medical ED, but ED providers/social workers could utilise on-call MH staff if questions. Paediatric psychology + psychiatric consultation services accessible in wider hospital. | Inpatients on the care pathway had significantly shorter LOS **(h2 5 0.06)** and fewer subspecialty consults than the C group (odds ratio 0.24 [95% CI 0.10–0.54]). |
| Majahan 2007  (US) | Child guidance team model implemented in PED. 24/7 staffing by master's level psychiatric social worker + board-certified child psychiatrist. Regardless of payer, the social worker, under the supervision of the child psychiatrist, evaluates all children with MH needs on-site. All children visiting for mental disorders (VMD, as determined by the evaluating ED clinician) are referred to the child guidance team for disposition. | Average LOS of VMD visits longer than non-VMD visits (236.04 minutes vs 134.69 minutes, mean difference 101.34, p=0.0001). LOS reduced significantly after program initiated (from 259.49 mins to 216.39 mins, mean difference 43.10 minutes, p=0.0001). Multiple regression found presence of child guidance team had the greatest effect in reducing mean LOS minutes by 44.23, p=0.00. |
| McGrady 2018 (CAN) | Paediatric psychology visits to CYP awaiting stem cell transplant (SCT), defined as individual/family therapy provided by licensed paediatric clinical psychologist. Following initial referral, triage, and assessment (typically completed within 24 hours post-referral), the psychologist then communicated diagnostic impressions, planned course of psychological treatment, and treatment goals to the team. Throughout the course of treatment, the psychologist worked closely with the psychosocial team (i.e., social work, child life, pastoral). | After controlling for demographics (age, gender, race, insurance status), clinical characteristics (SCT type, diagnosis), and number of paediatric psychology visits and year of psychology consultation, longer time to initial consultation significantly predicted a longer length of stay (p<.001). Each day increase in the time to consultation was associated with a 0.7% increase in the length of the initial SCT hospitalization when the effects of covariates were held constant. |
| McNicholas 2021 (IRE) | Hospitals 1+ 2: medical model PMHL services provided by psychiatry and MH nursing staff, with ad-hoc input from other hospital teams. PMHL team primarily supports the ED and wards, with very little support available for out-patient work. Hospital 1: 9am-5pm Mon-Fri service with morning cover at the weekends. Hospital 2: 9am-7pm weekend cover. Hospital 3 has 24 hour on-call service and sees young people using an MDT approach. Emergency and non-urgent referrals accepted; outpatient work offered. | Statistically significant difference noted between length of stay for patients admitted at the different hospitals (p=<0.001). Hospital 1 (mean length 6.67 hours), Hospital 2 (mean length 1.27 hours) Hospital 3 (mean length 2.13 hours). |
| Nagarsekar 2021 (AUS) | New KALM ED care pathway = MH assessment by paediatric or emergency medicine registrar (HEADSS assessment tool + suicide/other risks assessment) + consult with on-call psychiatrist to develop management plan. Medic could refer to MH clinicians if unforeseen complexities emerged or psychiatrist advised. Patients using the new pathway were assessed by fewer clinician, there was no duplication of the initial assessment, and no waiting time for a specialist MH clinician. TAU pathway = MH assessment was conducted by a MH clinician. | Median hours in ED. KALM = 4.13 (range 0.46–11.55), TAU= 5.09 (range 0.21–19.12). Adjusted for arrival during normal hours, KALM had median LOS of 58 min (p=0.055, 95% CI −119, 1.7), less than TAU. 56% of patients on the new pathway _+_ 63% on TAU breached 4h department target. Adjusted for arrival during normal hours, odds of breach were 32% less for KALM than TAU (OR 0.68, 95% confidence interval 0.36–1.31), although this was not statistically significant (P = 0.25). |
| Parker 2003  (CAN) | Child psychiatrist providing rapid response model: (a) Emergency, immediate consultation by child psychiatrist in ED/by phone. (b) Urgent consultation (daily urgent appointments for ED and community service providers in the working week, for patients not at immediate risk of harm to self to be booked directly into outpatient psychiatry appointments rather than be seen at the ED. (c) Education of providers who might use the service. | In multivariate analysis, the presence of the rapid response model was positively associated with; ED admissions (p=<0.05), non-ED admissions (p=<0.05); ED contacts without admission (p=<0.05) and average psychiatric LOS (p=<0.05), with presence or absence affecting all aside from ED contacts without admission. |
| Sheridan 2015  (US) | MDT including child psychiatrist + paediatric MH social worker devoted to CYP in PED with MH needs. Team staffed by the same psychiatrist and social worker to provide continuity of care for patients + ED team. Direct therapy and interventions provided in the PED (not described) + 1-month social worker outpatient follow-up to support transition to appropriate outpatient MH providers | Median PED LOS was 24.0 h preintervention and 20.9 h postintervention This was a decrease of 27% and was determined to be statistically significant (95% CI 0–46%; p = 0.05). |
| Uspal 2016  (US) | Dedicated MH team in ED 24/7 to provide: MH evaluations, care coordination, behavioural interventions, brief psychoeducation to patients/families. Team = a mental health evaluator (MHE) and a paediatric mental health specialist (PMHS). MHE is psychiatric-trained nurse/master’s degree social worker. PMHS is a bachelor’s degree–level provider who provides discharge planning and crisis management + monitor and helps manage patient behaviours in the ED. The physical environment of the ED was also adjusted to enhance safety**.** | A significant decrease in mean ED LOS was observed postintervention, from 332 minutes (95% confidence interval [CI] = 309–353 minutes) to 244 minutes (95% CI = 233–254 minutes). This change was sustained over time. On LOS subanalysis based on patient time of presentation, there was a significant decrease in mean LOS from 7 AM to 3 PM, 3 PM to 11 PM, and 11 PM TO 7 AM. Median LOS significantly decreased from 3 PM to 11 PM and 11 PM to 7 AM |
